# Effects of ordered mutations on dynamics in signaling networks

**DOI:** 10.1186/s12920-019-0651-z

**Published:** 2020-02-20

**Authors:** Maulida Mazaya, Hung-Cuong Trinh, Yung-Keun Kwon

**Affiliations:** 1 School of IT Convergence, University of Ulsan, 93 Daehak-ro, Nam-gu, Ulsan, 44610 Republic of Korea; 2 Faculty of Information Technology, Ton Duc Thang University, Ho Chi Minh City, Vietnam

**Keywords:** Ordered-mutations, Mutation-sensitivity, Order-specificity, Boolean dynamics, Signaling networks

## Abstract

**Background:**

Many previous clinical studies have found that accumulated sequential mutations are statistically related to tumorigenesis. However, they are limited in fully elucidating the significance of the ordered-mutation because they did not focus on the network dynamics. Therefore, there is a pressing need to investigate the dynamics characteristics induced by ordered-mutations.

**Methods:**

To quantify the ordered-mutation-inducing dynamics, we defined the mutation-sensitivity and the order-specificity that represent if the network is sensitive against a double knockout mutation and if mutation-sensitivity is specific to the mutation order, respectively, using a Boolean network model.

**Results:**

Through intensive investigations, we found that a signaling network is more sensitive when a double-mutation occurs in the direction order inducing a longer path and a smaller number of paths than in the reverse order. In addition, feedback loops involving a gene pair decreased both the mutation-sensitivity and the order-specificity. Next, we investigated relationships of functionally important genes with ordered-mutation-inducing dynamics. The network is more sensitive to mutations subject to drug-targets, whereas it is less specific to the mutation order. Both the sensitivity and specificity are increased when different-drug-targeted genes are mutated. Further, we found that tumor suppressors can efficiently suppress the amplification of oncogenes when the former are mutated earlier than the latter.

**Conclusion:**

Taken together, our results help to understand the importance of the order of mutations with respect to the dynamical effects in complex biological systems.

## Background

In a tumor cell, DNA damage restoration can have some errors such as chromosome abnormalities or genetic instability that result in a sequence of mutations [1]. The accumulated mutations can cause tumorigenesis or cancer development [2, 3]. Interestingly, this process can be affected by the order of genes subject to mutations. For example, it was observed in patients with *Myeloproliferative Neoplasms* that *JAK2* mutation followed by *TET2* mutation influenced the clinical features [3]. In another study [4], it has been shown that the timing of the *DNMT3A* mutation can affect the phenotypes of myeloid diseases in different ways. It was also found that the mutation of the *CSF3R* gene arising in the early severe congenital neutropenia stage is crucial to leukemia transformation [5]. Moreover, the mutation order influences the mutagen target size in tumor evolution [6] and results in complications in cancer biology [7].

Based on these observations, many methods attempted to investigate the effects of the order of mutation occurrence on cancer development [8], differences in clinical presentation [3], or response to targeted therapy [9] through biological experiments. In addition, statistical approaches were also developed to estimate the effect of the mutation order [10–12]. The understanding of the significance of ordered mutations can be enhanced through the analysis of biological networks. For example, a few previous studies reported that specific sequences of mutations which are efficient in cancer development were related to gene-gene interaction networks [13–15]. In addition, simple network structural characteristics were found to be relevant to the ordered mutations [16, 17]. Other studies also implicitly attempted to relate the dependency of ordered compensatory perturbation with a growth rate of cells in metabolic systems, through the network structural analysis and biological experiments [18–20]. Although these previous studies explained the significance of the ordered mutations, the results are limited due to the deficiency of analysis on the network dynamics.

Accordingly, we aim to investigate interesting dynamical characteristics induced by the ordered double-mutations in the signaling networks. To this end, we employed a Boolean network model and defined two measures with respect to ordered-mutation-inducing dynamics, the mutation-sensitivity and the order-specificity, against a double knockout mutation. The former represents how likely a network state trajectory changes by an ordered mutation whereas the latter indicates the likelihood that the network state trajectories induced by different orders of a mutation are not identical. Through intensive investigations in three real signaling networks, we found that a network is more sensitive when a double knockout mutation occurs in the order to induce a longer path and a smaller number of paths than in the reverse order. In addition, the existence of a feedback loop structure reduced the mutation-sensitivity as well as the order-specificity. Next, we investigated the ordered-mutation-inducing dynamics of some functionally important genes such as drug-targets, tumor suppressors, and oncogenes. It was interesting that the number of drug-targets subject to mutations was negatively correlated to the mutation-sensitivity, whereas the mutation order was more specific in mutations in drug-targets than non-drug-targets. In addition, both the mutation-sensitivity and the order-specificity of same-drug-targets were higher than those of the different-drug-targets, respectively. Furthermore, we found that tumor suppressors can efficiently suppress the amplification of oncogenes when the former genes are mutated earlier than the latter genes. Taken together, our results enhance the understanding of the dynamical effects of ordered double-mutations in complex biological systems.

## Methods

### Datasets

In this work, we employed datasets of three molecular interaction networks, a human cancer signaling (HCS) network with 1192 genes and 3102 interactions constructed in previous studies [21, 22] to provide a map of human cancer signaling, another large-scale signaling network with 1659 genes and 7964 interactions constructed in previous study [23] which was derived from the Kyoto Encyclopedia of Genes and Genomes database [24] (KEGG) network, and a *T-cell large granular lymphocyte* survival signaling [16, 25, 26] (TGL) network with 61 genes and 193 interactions about the long-term survival of competent cytotoxic *T lymphocytes* in humans. Moreover, we retrieved lists of drug-targets, tumor suppressors, and oncogenes from the DrugBank [27], TSGene [28, 29], and ONGene [30] databases, respectively. Accordingly, we found 504, 538, and 20 drug-targets in the HCS, KEGG, and TGL networks, respectively. In addition, we identified 245 tumor-suppressors and 227 oncogenes in HCS, 176 tumor-suppressors and 168 oncogenes in KEGG, and 6 tumor-suppressors and 13 oncogenes in TGL networks (see Additional file 1: Table S1, S2 and S3 for the lists of drug-targets, tumor suppressor genes, and oncogenes in HCS, KEGG, and TGL networks, respectively).

### A Boolean network model

To analyze the network dynamics induced by ordered mutations, we applied a Boolean network model, which is the simplest computational model [31–33] and has been used to examine complex behaviors of biological networks [34, 35]. A Boolean network is represented by a directed graph *G*(*V*, *A*) where *V* = {*v*_1_, *v*_2_, …, *v*_*N*_} is a set of nodes and *A* ⊆ *V* × *V* is a set of directed links. Each *v*_*i*_ ∈ *V* has a value of 1 (on) or 0 (off), which indicates the possible states of the corresponding elements. A directed link (*v*_*i*_, *v*_*j*_) represents a positive (activating) and a negative (inhibiting) relationship from *v*_*i*_ to *v*_*j*_. Let *v*(*t*) denote the state of node *v* at time-step *t* (*t* is a non-negative integer). When a state of *v*_*i*_ at time *t* + 1 is determined by the values of *k*_*i*_ and other nodes ${v}_{i_1},{v}_{i_2},\dots, {v}_{i_{k_i}}$ with a link to *v*_*i*_ at time *t*, then the update rule of *v*_*i*_ is represented by a Boolean function ${f}_i:{\left\{0,1\right\}}^{k_i}\to \left\{0,1\right\}$. Herein, all nodes are synchronously updated, and we employed a nested canalyzing function (NCF) model [36, 37] to describe an update rule *f*_*i*_ as follows:

$$ {f}_i\left({v}_{i_1}(t),{v}_{i_2}(t),\dots, {v}_{i_{k_i}}(t)\right)=\left\{\begin{array}{c}\begin{array}{cc}{O}_1\kern1em & if\ {v}_{i_1}(t)={I}_1\kern19.25em \\ {}{O}_2\kern1em & if{v}_{i_1}(t)\ne {I}_1\ \mathrm{and}\ {v}_{i_2}(t)={I}_2\kern11.25em \\ {}{O}_3\kern1em & if\ {v}_{i_1}(t)\ne {I}_1\ \mathrm{and}\ {v}_{i_2}(t)\ne {I}_2\ \mathrm{and}\ {v}_{i_3}(t)={I}_3\kern3em \end{array}\\ {}\vdots \\ {}\begin{array}{cc}{O}_{k_i}\kern0.75em & if\ {v}_{i_1}(t)\ne {I}_1\cdots {v}_{i_{k_i-1}}(t)\ne {I}_{k_i-1}\ \mathrm{and}\ {v}_{i_{k_i}}(t)={I}_{k_i}\\ {}{O}_{def}& otherwise\kern20.75em \end{array}\end{array}\right. $$

where *I*_*m*_ and *O*_*m*_ (*m* = 1, 2, …, *k*_*i*_) represent the canalyzing and canalyzed Boolean values, respectively, and *O*_*def*_ is set to $1-{O}_{k_i}$ in general. Unfortunately, it is not easy to infer the canalyzing and the canalyzed values in the real signaling networks, so we specified *I*_*m*_ and *O*_*m*_ values independently and uniformly at random between 0 and 1. We note that NCFs have been shown to properly fit real biological experimental data [36, 38], and many biological networks were successfully simulated by NCFs [39, 40].

A *network state* at time *t* can be denoted by a list of state values of all nodes, **v**(*t*) = [*v*_1_(*t*), *v*_2_(*t*), …, *v*_*N*_(*t*)] ∈ {0, 1}^*N*^. Every network state transits to another network state determined by a set of Boolean update functions *F* = {*f*_1_, *f*_2_, …, *f*_*N*_} which is synchronously updated f_1_, f_2_, …f_N_, and f_1_, f_2_, …f_N_ eventually converges to either a fixed point or a limit-cycle attractor. The *attractor* is rigorously defined as follows.

***Definition.*** Let **v**(0), **v**(1), ⋯**v**(*t*), ⋯ be a network state trajectory starting at **v**(0). Then, the *attractor* denoted by 〈*G*, *F*, **v**(0)〉 is represented by an ordered finite sub-list of the trajectory, [**v**(*τ*), **v**(*τ* + 1), …, **v**(*τ* + *p* − 1)], where *τ* is the smallest time-step such that **v**(*t*) = **v**(*t* + *p*) for ∀*t* ≥ *τ* with **v**(*i*) ≠ **v**(*j*) for ∀ *i* ≠ *j* ∈ {*τ*, *τ* + 1, …, *τ* + *p* − 1}. Herein, *p* is called the attractor length.

To identify an attractor, the network state trajectory is computed by synchronously updating the state values of all nodes until the time-step *t* is found such that **v**(*t*) = **v**(*t* + *p*).

### Computation of mutation-sensitivity and order-specificity

Given a Boolean network *G*(*V*, *A*) with a set of nodes *V* = {*v*_1_, *v*_2_, …, *v*_*N*_} specified by a set of corresponding update-rules *F* = {*f*_1_, *f*_2_, …, *f*_*N*_}, consider a state trajectory starting from an arbitrary initial state. When a network is subject to a mutation, the trajectory may converge to a different attractor. Then, the network is regarded as sensitive to the mutation. Let *W* ⊆ *V* be a set of genes subject to knockout mutations [41, 42], and we denote by *F*^*W*^ as a set of update-rules where every gene in *W* ⊆ *V* is frozen to 0 (off state) in *F*. In this work, we investigate the effect of the ordered double-mutations on the network dynamics. Let (*v*_*k*_, *v*_*l*_) be an ordered pair of nodes subject to a double-mutation with a time gap *T*, which means that *v*_*k*_ is first mutated at time-step *t* = 0, and then *v*_*l*_ is mutated at time-step *t* = *T*. In other words, the time gap represents the time-step lag between the occurrences of the first and the second mutation. We can implement it by assuming that ${F}^{\left\{{v}_k\right\}}$ and ${F}^{\left\{{v}_k,{v}_l\right\}}$ are effective for 0 ≤ *t* < *T* and *t* ≥ *T*, respectively. It has been known that the notion of the time gap is important since it can affect the mutation process [14, 43, 44]. Then, when we denote by ${F}_{\left({v}_k,{v}_l\right)}^{\prime }$ a series of sets of the update rules by (*v*_*k*_, *v*_*l*_)-ordered double-mutation, we can define the mutation-sensitivity as follows:

$$ \delta =\frac{\sum_{\mathbf{v}(0)\in S}I\left(\left\langle G,F,\mathbf{v}(0)\right\rangle \ne \left\langle G,{F}_{\left({v}_k,{v}_l\right)}^{\prime },\mathbf{v}(0)\right\rangle \right)}{\left|S\right|},\kern0.5em (1) $$

where *S* is a set of considered initial-states and *I*(*condition*) denotes an indicator function that returns 1 if the *condition* is true and 0, otherwise. In other words, *δ* represents the probability that a network converges to a different attractor by the double knockout mutation. To quantify the specificity of dynamics with respect to the mutation order, we define the order-specificity as follows:

$$ \Delta =\frac{\sum_{\mathbf{v}(0)\in S}I\left(\left\langle G,{F}_{\left({v}_k,{v}_l\right)}^{\prime },\mathbf{v}(0)\right\rangle \ne \left\langle G,{F}_{\left({v}_l,{v}_k\right)}^{\prime },\mathbf{v}(0)\right\rangle \right)}{\left|S\right|}.(2) $$

**Fig. 1:**
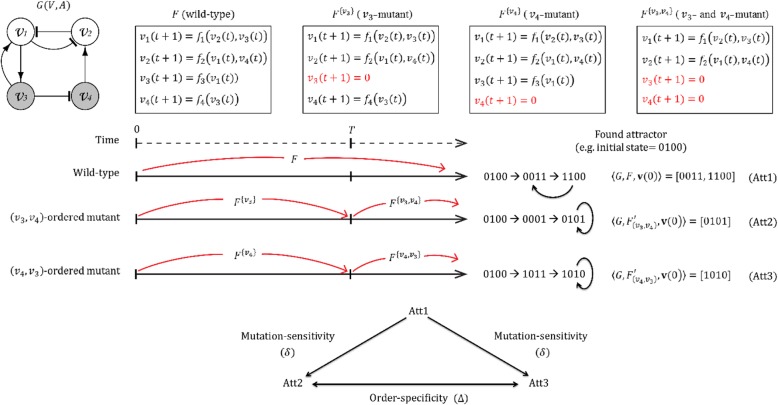
An example of mutation-sensitivity and order-specificity computation. Given a network *G*(*V*, *A*) with a set of wild-type update rules *F*, let *v*_3_ and *v*_4_ be a pair of nodes subject to a double-mutation with a time gap *T*, and 0100 ∈ *S* an initial state. *F*^*W*^ denotes a *W*-mutant update rule set where every gene in *W* ⊆ *V* is frozen. In this example, the wild-type attractor (Att1) is computed by applying *F* all the time. On the other hand, a (*v*_3_, *v*_4_)-ordered (or (*v*_4_, *v*_3_)-ordered) mutant attractor denoted by Att2 (resp. Att3) is computed by assuming that ${F}^{\left\{{v}_3\right\}}$ (resp. ${F}^{\left\{{v}_4\right\}}$) and ${F}^{\left\{{v}_3,{v}_4\right\}}$ apply for 0 ≤ *t* < *T* and *T* ≤ *t*, respectively. The mutation-sensitivity of (*v*_3_, *v*_4_)-ordered (or (*v*_4_, *v*_3_)-ordered) double-mutation is computed by comparing Att1 and Att2 (resp. Att3). The order-specificity is computed by comparing Att2 and Att3

In other words, Δ represents the probability of a network converging to different attractors by different mutation orders. Figure 1 shows an illustrative example of the mutation-sensitivity and the order-specificity notions. Let {*v*_3_, *v*_4_} be a pair of genes subject to mutations and 0100 be a given initial state. Then, a wild-type attractor denoted by Att1 is computed by applying *F* all the time. Next, we compute Att2 and Att3 to which the network converges against (*v*_3_, *v*_4_)- and (*v*_4_, *v*_3_)-ordered mutations, respectively. Note that ${F}^{\left\{{v}_3\right\}}$ (or ${F}^{\left\{{v}_4\right\}}$) and ${F}^{\left\{{v}_3,{v}_4\right\}}$ applies for 0 ≤ *t* < *T* and *t* ≥ *T*, respectively, in computing Att2 (resp. Att3). When Att2 (or Att3) is not identical to Att1, the network is regarded as sensitive to the (*v*_3_, *v*_4_)-ordered (resp. (*v*_4_, *v*_3_)-ordered) mutation. In addition, the network dynamics are specific to the mutation order if Att2 and Att3 are not identical to each other. We note that a pair of genes (*v*_*k*_, *v*_*l*_) with a common child node in the network are excluded from analysis in this study. In case that there exists such a common child node *v*_*c*_, it is probable that the update of *v*_*c*_ is differently affected by the (*v*_*k*_, *v*_*l*_)-ordered and the (*v*_*l*_, *v*_*k*_)-ordered mutations, because the occurrence order of *v*_*k*_ and *v*_*l*_ in the NCF to update *v*_*c*_ represents the degree of influence on the update of *v*_*c*_. Finally, we note that the network dynamics can depend on the initial network states. Therefore, a total of 1000 initial-states (i.e., |*S*| = 1000) were randomly generated to compute the mutation-sensitivity and the order-specificity values in Eqs. (1) and (2) in all simulations of this study.

### Structural characteristics of ordered gene pairs

Some structural characteristics of genes are related to network dynamic stability [45]. In this study, we considered the following structural properties to investigate the relationship between ordered mutations.

- The path length of (*v*_*i*_, *v*_*j*_) denoted by *l*(*v*_*i*_, *v*_*j*_) is defined by the number of links included in the shortest path from *v*_*i*_ and *v*_*j*_.
- The number of paths of (*v*_*i*_, *v*_*j*_) denoted by *n*(*v*_*i*_, *v*_*j*_) is defined by the number of non-identical paths from *v*_*i*_ and *v*_*j*_.
- A feedback loop (FBL) is a sequence of nodes where no node is repeated except the starting and the ending nodes. It has been known that FBLs play an important role in controlling dynamics behaviors of cellular signaling networks [32, 46, 47].

### Random network generation

To verify that the results of mutation-sensitivity and order-specificity in the real molecular interaction networks are consistent with randomly structured networks, we generated random networks using the Barabási Albert (BA) [48] model which is a kind of network growth model with a preferential attachment scheme.

### Parallel computation

For efficient *in-silico* simulations, we basically implemented the program code using PANET [35] which is an analysis tool of the network dynamics analysis tool using the OpenCL library. This enables us to compute a large number of attractors in parallel by assigning each initial random state in Eqs. (1) and (2) to a processing unit of CPUs and/or GPUs.

### Statistical analysis

In this paper, we conducted the Mann-Whitney U test to see if the mutation-sensitivity and the order-specificity are significantly different between any two groups, because they are not normally distributed. The Mann-Whitney U test combines two groups and ranks them. Then, it calculates a statistic of the difference of the rank sum between two resampled groups. We used MedCalc [49] Statistical Software (version 13.0.6) for the Mann-Whitney U test.

## Results

In this study, we simulated the ordered-mutation-inducing dynamics of three real biological networks using the Boolean network model (see Methods for details). As explained in Methods section, we note that a total of 1000 initial-states were randomly generated to compute the mutation-sensitivity and the order-specificity values in Eqs. (1) and (2) in all simulations. In addition, we constructed a set of ordered gene pairs to be investigated, *Ω*, for tractable simulation. It consists of all ordered pairs of genes in the case of TGL network with a small number of nodes (|*N*| = 61), whereas 30,000 randomly selected gene pairs in the case of large-scale networks of HCS (|*N*| = 1192) and KEGG (|*N*| = 1659). Considering the different network size, we also set the time gap (*T*) to 2–20 in HCS and KEGG, and 1–10 in TGL, respectively.

### Distributions of ordered-mutation-inducing dynamics

**Fig. 2:**
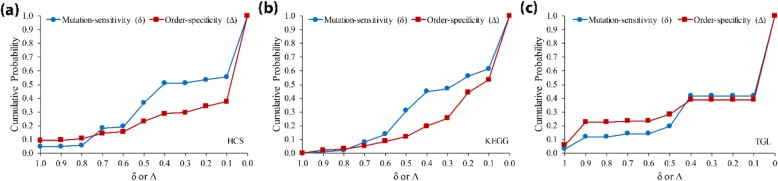
Cumulative probability distributions of mutation-sensitivity and order-specificity values in signaling networks. Mutation-sensitivity and order-specificity of ordered gene pairs were examined. (**a-c**) Results in HCS, KEGG, and TGL, respectively. The time gap (*T*) was set to 20 in (**a**) and (**b**), and 10 in (**c**)

To see how frequently the network dynamics are affected by ordered mutations, we examined the accumulative distributions of the mutation-sensitivity (*δ*) and order-specificity (*Δ*) values of examined gene pairs (*Ω*) in three signaling networks (Fig. 2) in the case of the largest time gap (i.e., *T* = 20 for HCS and KEGG, and *T* = 10 for TGL). In the figure, the *y*-axis value means the cumulative probability of mutation-sensitivity or order-specificity larger or equal to the *x*-axis value. We observed that the cumulative probabilities of *δ* ≥ 0.1 in HCS, KEGG, and TGL were 0.56, 0.62, and 0.39, respectively. This implies that it is not rare to observe that the network dynamics are sensitive against the double-mutations. It was also observed that the cumulative probabilities of *Δ* ≥ 0.1 in HCS, KEGG, and TGL were 0.38, 0.54, and 0.32, respectively. We need to note that an order-specificity of zero can be observed even in the case of gene pairs with nonzero mutation-sensitive values according to the definitions. Therefore, the observed distribution of the order-specificity implies that the mutation order is considerably critical to the network dynamics.

### Relation between structural characteristics and ordered-mutation-inducing dynamics

**Fig. 3:**
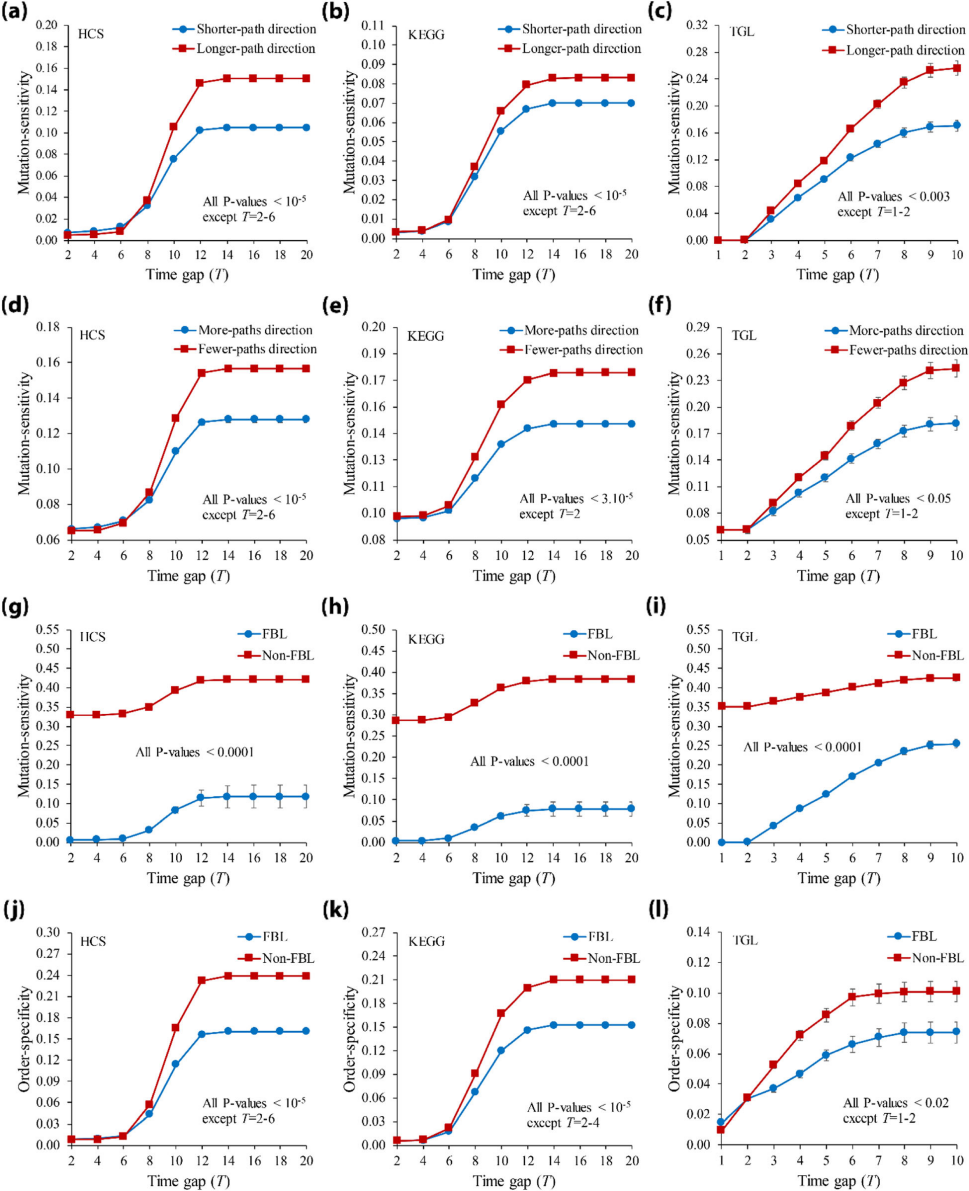
Relations of structural properties with the ordered-mutation-inducing dynamics in signaling networks. (**a-c**) Mutation-sensitivity results with respect to the path length in HCS, KEGG, and TGL, respectively. All pairs of nodes involving an FBL were classified into ‘Shorter-path direction’ and ‘Longer-path direction’ groups where *l*(*v*_*i*_, *v*_*j*_) is smaller and larger than *l*(*v*_*j*_, *v*_*i*_), respectively. (**d-f**) Mutation-sensitivity results with respect to the number of paths in HCS, KEGG, and TGL, respectively. All pairs of nodes were classified into ‘More-paths direction’ and ‘Fewer-paths direction’ groups such that *n*(*v*_*i*_, *v*_*j*_) is smaller and larger than *n*(*v*_*j*_, *v*_*i*_), respectively. (**g-i**) Mutation-sensitivity results with respect to of the FBLs in HCS, KEGG, and TGL, respectively. All pairs of nodes were classified into ‘FBL’ and ‘Non-FBL’ groups such that any gene in the pair is involved in an FBL or not. (**j-l**) Order-specificity results with respect to the feedback loops in HCS, KEGG, and TGL, respectively. Time gap (*T*) was set to 2–20 in HCS and KEGG networks, and 1–10 in TGL networks. The error bar represents the standard error deviation

There have been many previous studies on the relationship between the structural properties and the dynamical behavior in biological networks [45, 50, 51]. Inspired by them, we investigated the relationships between some structural properties and ordered-mutation-inducing dynamics (Fig. 3). We first classified every ordered gene pair (*v*_*i*_, *v*_*j*_) in *Ω* into ‘Shorter-path direction’ and ‘Longer-path direction’ groups if *l*(*v*_*i*_, *v*_*j*_) < *l*(*v*_*j*_, *v*_*i*_) and *l*(*v*_*i*_, *v*_*j*_) > *l*(*v*_*j*_, *v*_*i*_) (see Methods for the definition), respectively, and forced knockout mutations in the order of *v*_*i*_ and *v*_*j*_. We note that the gene pair (*v*_*i*_, *v*_*j*_) which is not bidirectionally connected was excluded from analysis to remove the effect of the connectedness factor on the dynamics. We compared the average mutation-sensitivity values between them (Fig. 3(a)-(c); all *P*-values using the Mann-Whitney U test). As shown in the figure, the mutation-sensitivity of the ‘Longer-path direction’ group is significantly higher than that of the ‘Shorter-path direction’ group in all signaling networks for most time gap parameter values. In other words, the network is more sensitive when the double knockout mutation occurs in the order inducing a longer path than in the reverse order. Next, we classified every ordered gene pair (*v*_*i*_, *v*_*j*_) into ‘More-paths direction’ and ‘Fewer-paths direction’ groups if *n*(*v*_*i*_, *v*_*j*_) > *n*(*v*_*j*_, *v*_*i*_) and *n*(*v*_*i*_, *v*_*j*_) < *n*(*v*_*j*_, *v*_*i*_) (see Methods for the definition), respectively, and forced knockout mutations in the order of *v*_*i*_ and *v*_*j*_. We compared the average mutation-sensitivity values between them (Fig. 3(d)-(f); all *P*-values using the Mann-Whitney U test). As shown in the figure, the mutation-sensitivity of the former group is significantly smaller than that of the latter group in both signaling networks, almost irrespective of the time gap parameter. In other words, the network is more sensitive when the double knockout mutation occurs in the order involving fewer paths than in the reverse order. We note that our previous study showed that the dynamics influence from a gene on another gene is likely to be lessened as the path length increases and the number of paths decreases [50]. Thus, it is interesting that both the ‘Longer-path direction’ and ‘Fewer-paths direction’, which showed relatively higher mutation-sensitivity values, represent ways to induce a smaller dynamics-influence from the first mutated gene on the second mutated gene than the reverse order. Finally, we considered the FBL as another interesting structural property for investigation, because many previous studies have proven the relation of it with the dynamical behavior of biological networks [46, 47, 52]. We classified every ordered gene pair into ‘FBL’ and ‘Non-FBL’ groups if any gene in the pair is involved in an FBL or not, respectively. Then, we compared the average mutation-sensitivity values between them (Fig. 3(g)-(i); all *P*-values using the Mann-Whitney U test). As shown in the figure, the mutation-sensitivity of the former group is significantly smaller than that of the latter group in both signaling networks regardless of the time gap parameter. In addition, we further compared the order-specificity between the two groups (Fig. 3(j)-(l)). (Note that it is not feasible to compare the order-specificity between Longer- and Shorter-path direction groups, or More- and Fewer-paths direction groups because the relation of ordered gene pair in each group is not symmetric). We found that the order-specificity of the FBL group was significantly smaller than that of the Non-FBL group. This implies that the FBL structure reduced the specificity of the mutation order. Taken together, we can conclude that ordered-mutation-inducing dynamics are highly related with the structural properties such as the path length, the number of paths, and the FBL. In addition, we investigated the relationship between these structural properties and the ordered-mutation-inducing dynamics in a numbers of BA random networks (see Methods section) and found consistent results (see Additional file 1: Figure S1). In other words, our findings might be observed in networks with various structures. Finally, the results can be related with a recent study about the occurrence of different cancer types by the mutation order [53]. The authors in that study found that a double mutation in the order of *EP300* and *TP53* genes was relatively frequent in patients with esophageal and bladder urothelial carcinoma. On the other hand, the mutation in the reverse order was enriched in patients with cervical squamous cell carcinoma and endocervical adenocarcinoma. It is intriguing that the order of *EP300* and *TP53* belongs to ‘Shorter-path direction’, ‘More-paths direction’, and ‘FBL’ groups in the HCS network according to our classification, all of which indicated a relatively low mutation-sensitivity.

### Analysis of drug-target genes with respect to ordered-mutation-inducing dynamics

**Fig. 4:**
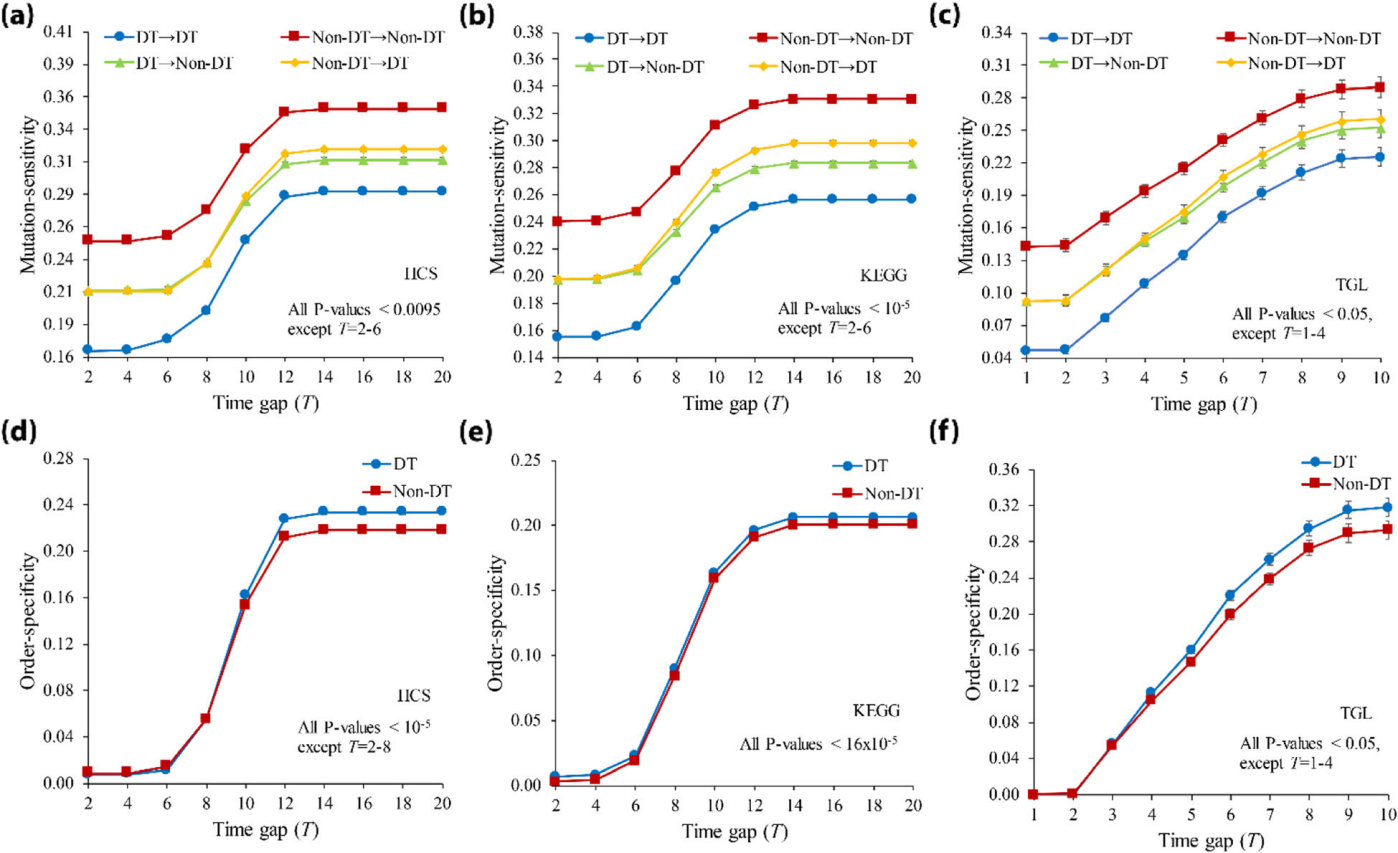
Analysis of ordered-mutation-inducing dynamics with respect to drug-targets in signaling networks. (**a-c**) Mutation-sensitivity results in HCS, KEGG, and TGL, respectively. All genes were specified by ‘Drug-target (DT)’ and ‘Non-drug-target (Non-DT)’, and every gene pair was classified into four groups, ‘DT → DT’, ‘DT → Non-DT’, ‘Non-DT → DT’, and ‘Non-DT → Non-DT’. (**d-f**) Order-specificity results in HCS, KEGG, and TGL, respectively. Time gap (*T*) was set to 2–20 in HCS and KEGG networks, and 1–10 in TGL networks. The error bar represents the standard error deviation

**Fig. 5:**
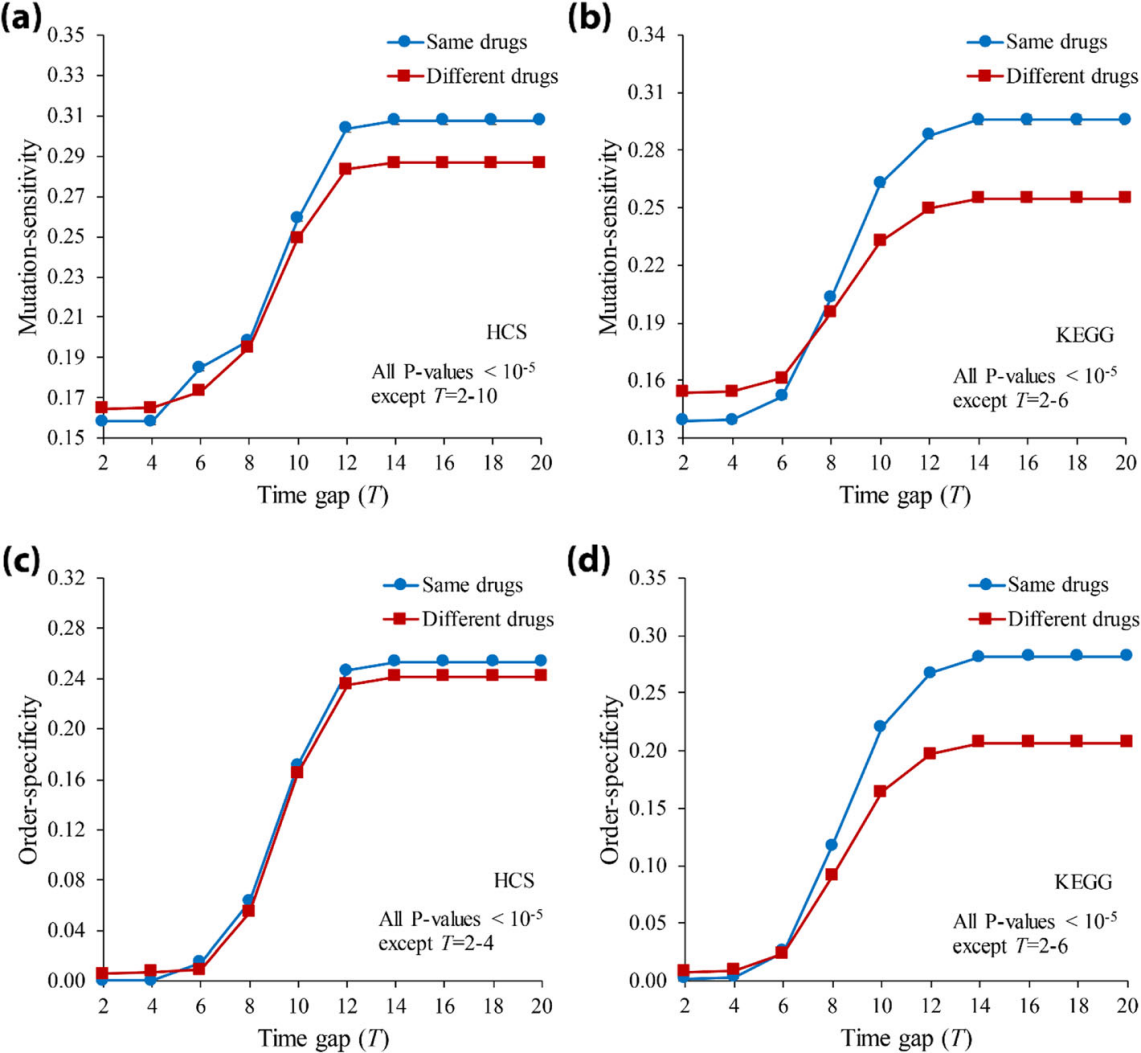
Comparison of ordered-mutation-inducing dynamics between gene pairs targeting same and different drugs in signaling networks. All pairs of genes were classified into ‘Same-drug’ or ‘Different-drug’ groups if the two genes in a pair target a same drug or different drugs, respectively. (**a-b**) Mutation-sensitivity results in HCS and KEGG, respectively. (**c-d**) Order-specificity results in HCS and KEGG, respectively. All time gap (*T*) was set to 2–20. The error bar represents the standard error deviation. Note that TGL network was excluded from analysis, because there was no pair of genes belonging to ‘Same-drug’ group

Some previous studies have investigated the characteristics of drug-target genes through network-based structural analysis [54, 55], and the findings were useful to understand tumorigenesis in cancer [56]. In this study, we extended it to dynamic analysis by investigating the ordered-mutation-inducing dynamics of drug-targets in signaling networks. We first specified all genes as ‘Drug-target (DT)’ and ‘Non-drug-target (Non-DT)’ genes (see Methods and Additional file 1: Table S1, S2 and S3). Then, we classified every ordered gene pair in *Ω* into four groups: ‘DT → DT’, ‘DT → Non-DT’, ‘Non-DT → DT’, and ‘Non-DT → Non-DT’. After forcing double knockout mutations, we compared the average mutation-sensitivity value among them (Fig. 4(a)-(c); all *P*-values using the Mann-Whitney U test). As shown in the figure, the values of ‘Non-DT → Non-DT’ and ‘DT → DT’ groups were highest and lowest, and they were the bounds for the values of other groups. Furthermore, the sensitivity of the ‘Non-DT → DT’ group was significantly higher than that of the ‘DT → Non-DT’ group, for most time gap values. Considering that these two groups are identical to each other except for the order in a gene pair, the result implies that the sensitivity difference was caused by only the mutation order. We further examined the order-specificity values of DT and Non-DT groups (Fig. 4(d)-(f); all *P*-values using the Mann-Whitney U test) and found that the former is larger than the latter. This finding is interesting considering that the mutation-sensitivity of ‘DT → DT’ was smaller than that of ‘Non-DT → Non-DT’ in Fig. 4(a)-(c). In other words, the network is less sensitive, but the mutation order is more critical when drug-target genes are mutated than when non-drug-target genes are mutated. Moreover, we further investigated the ‘DT → DT’ group by classifying every gene pair in the group into ‘Same drug’ and ‘Different drug’ sub-groups (see Additional file 1: Table S1, S2 and S3) for cases where both genes of a pair are targeted using the same drug or different drugs, respectively (TGL network was excluded from this analysis because there is no pair of genes belonging to ‘Same drug’ group). We found that both the mutation-sensitivity and order-specificity of the ‘Different drug’ group were less than those of the ‘Same drug’ group (Fig. 5). This implies that the network is more sensitive, and the mutation order is more specific when drug-targets from the same-drug are mutated than when drug-targets from the different-drug are mutated. Interestingly, this observation can be linked to some previous experimental studies about multiple drug treatments. For example, a specific sequential treatment of *roscovitine* before *doxorubicin* is synthetically lethal in breast cancer cell [57] and the treatment order of double-drugs with the shared targets is significant to the treatment efficiency [58]. In addition, our result implies that the ordered-mutation-inducing dynamics can be useful to predict a new drug-target gene which may show relatively lower mutation-sensitivity and the higher order-specificity when it is subject to the ordered mutation with another drug-target gene together.

### Analysis of tumor suppressor and oncogenes with respect to ordered-mutation-inducing dynamics

**Fig. 6:**
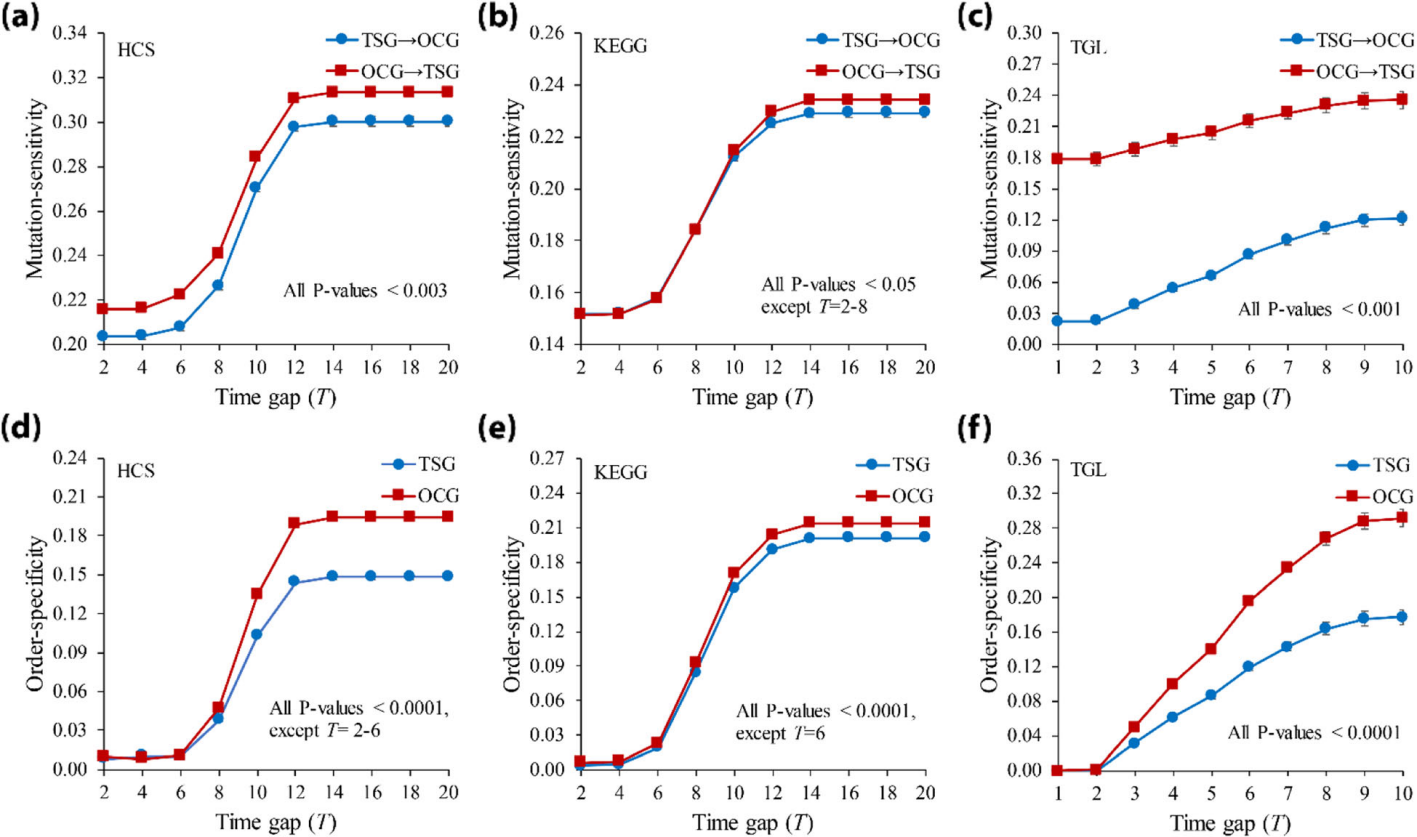
Analysis of ordered-mutation-inducing dynamics with respect to tumor suppressors and oncogenes in signaling networks. (**a-c**) Mutation-sensitivity results in HCS, KEGG, and TGL, respectively. All genes were specified by ‘Tumor suppressor gene (TSG)’ and ‘Oncogene (OCG)’ groups, and every ordered gene pair was classified into ‘TSG → OCG’ and ‘OCG → TSG’ groups. (**d-f**) Order-specificity results in HCS, KEGG, and TGL, respectively. Time gap (*T*) was set to 2–20 in HCS and KEGG networks, and 1–10 in TGL networks. The error bar represents the standard error deviation

It is known that tumor suppressors and oncogenes perform their cellular functions jointly in tumor progressions [59, 60], and tumor suppressors can be considered as therapeutic targets for cancer drugs [61, 62]. In this study, we investigated the ordered-mutation-inducing dynamics of tumor suppressors and oncogenes in signaling networks. We first specified all genes in the networks as ‘Tumor suppressor genes (TSG)’ and ‘Oncogenes (OCG)’ (see Methods and Additional file 1: Table S1, S2 and S3), and then identified two groups of ordered gene pairs in *Ω*, ‘TSG → OCG’ and ‘OCG → TSG’. For every ordered pair of genes, we computed the mutation-sensitivity after forcing double knockout mutations according to the order of gene pair. Then, we compared the average mutation-sensitivity value between those two groups (Fig. 6(a)-(c); all *P*-values using Mann-Whitney U test). As shown in the figure, the mutation-sensitivity value of the former group was significantly smaller than that of the latter group in all signaling networks, almost irrespective of the time gap. In other words, the network is more sensitive when oncogenes were mutated before tumor suppressors than the reverse order. In addition, we further compared the order-specificity between two groups, ‘TSG’ and ‘OCG’, and found that the order-specificity values of the former group were smaller than the latter group, almost irrespective of the time gap (Fig. 6(d)-(f); all *P*-values were obtained using Mann-Whitney U test). This finding can be also related to some previous studies on the ordered mutations between oncogenes and tumor suppressor genes. For example, the double mutation in the order of *TP53* and *NOTCH*, which are representative tumor-suppressor and oncogenes, respectively, was frequently observed in early stage of esophageal carcinoma patients [53], whereas the reverse-ordered mutation is likely to lead to a metastasis progression in mouse experiments [63, 64]. It was also shown that alteration of *RAS*, which is another oncogene, before loss of *P53* formed a malignant tumor with metastatic behavior, but the reverse-ordered mutation resulted in benign tumors [2, 65].

## Discussion

In this study, we defined the mutation-sensitivity and the order-specificity based on a Boolean network model to unravel the effects of ordered mutations on dynamics in signaling networks. It was interesting to observe that some structural properties of signaling networks can be a good indicator to explain the dynamical behavior with respect to ordered-mutation experiments. In addition, it was shown that various functionally important genes are related to the ordered-mutation-inducing dynamic. These results can enhance the understanding of the dynamic effects of ordered double-mutations on complex dynamics of large-scale biological systems, which supports the usefulness of our approach. Despite the usefulness of our approach, there are some limitations to be discussed. In this study, we employed the random nested canalyzing function to simulate the Boolean dynamics of the molecular signaling networks. This artificial specification can be a limitation of this study, although some previous studies have proven the usefulness of the model in fitting the update rules from the real biological data [36, 38]. Another concern is the synchronous update scheme, which is less realistic than the asynchronous update scheme. Therefore, a future study will include an approach to more accurately model the update rule inferred from real biological data.

## Conclusions

Many previous studies investigated ordered mutations and found statistical relations with cancer development. Recently, these studies were extended to incorporate the analysis of biological networks. However, they are limited in identifying the significance of ordered mutations because they did not focus on analysis of the network dynamics. In this regard, we quantified the ordered-mutation-inducing dynamics by defining the mutation-sensitivity and the order-specificity measures using a Boolean network model. Specifically, they represent the probability that a network converges to a different attractor by a double knockout mutation, and the probability with which a network converges to different attractors by different mutation orders, respectively. It was not rare to observe both nonzero sensitivity and specificity values in large-scale signaling networks. In addition, we examined the relationship between the structural characteristics such as the path length, the number of paths, and the feedback loop with the ordered-mutation-inducing dynamics in the signaling networks. Interestingly, they showed significant relationships, which implies that such structural properties need to be considered in experimental studies with respect to ordered-mutation experiments. Next, we investigated the ordered-mutation-inducing dynamics of various functionally important genes. The numbers of drug-targets genes were negatively correlated to the mutation-sensitivity, whereas the network was more specific to the order of mutations subject to drug-targets genes than the rest genes. In addition, we found that tumor suppressors can efficiently suppress the amplification of oncogenes when the former genes are mutated earlier than the latter genes. Taken together, our results enhance the understanding of the dynamic effects of ordered double-mutations on complex dynamics of large-scale biological systems.

## Supplementary information

**Additional file 1 Figure S1.** Relations of structural properties with ordered-mutation-inducing dynamics in BA network. A total of 250 BA random networks with |*V*| = 50 and |*A*| = 100 were generated. The time gap (*T*) was set to 1–10. **(a)** Mutation-sensitivity result with respect to the shortest path length. All pairs of nodes involving an FBL were classified into ‘Shorter-path direction’ and ‘Longer-path direction’ groups according that *l*(*v*_*i*_, *v*_*j*_) < *l*(*v*_*j*_, *v*_*i*_) and *l*(*v*_*i*_, *v*_*j*_) > *l*(*v*_*j*_, *v*_*i*_), respectively. **(b)** Mutation-sensitivity result with respect to the number of paths. All pairs of nodes were classified into ‘More-paths direction’ and ‘Fewer-paths direction’ groups according that *n*(*v*_*i*_, *v*_*j*_) > *n*(*v*_*j*_, *v*_*i*_) and *n*(*v*_*i*_, *v*_*j*_) < *n*(*v*_*j*_, *v*_*i*_), respectively. **(c)** Mutation-sensitivity result with respect to the FBLs. All pairs of nodes were classified into ‘FBL’ and ‘Non-FBL’ groups, according that any gene of the pair is involved by an FBL or not. **(d)** Order-specificity result with respect to the FBLs. All *P*-values were computed using the Mann-Whitney U test. **Table S1.** Gene information of HCS consisting 1192 genes, including its association with drug-target, tumor suppressor, and oncogene. **Table S2.** Gene information of KEGG consisting 1659 genes, including its association with drug-target, tumor suppressor, and oncogene. **Table S3.** Gene information of TGL consisting 61 genes, including its association with drug-target, tumor suppressor, and oncogene.

## Data Availability

All data generated or analyzed during this study are included in this published article and its additional files.

## Abbreviations

BA: Barabási Albert
DT: Drug-target
FBL: Feedback-loop
HCS: Human cancer signaling
KEGG: Kyoto Encyclopedia of Genes and Genomes database
NCF: Nested canalyzing function
OCG: Oncogene
TGL: T-cell large granular lymphocyte
TSG: Tumor suppressor gene
