# Supplementary material for: Effects of ordered mutations on dynamics in signaling networks
Source: BMC Med Genomics. 2020 Feb 20;13(Suppl 4):13. doi: 10.1186/s12920-019-0651-z (PMC7032007; doi:10.1186/s12920-019-0651-z)
Supplement: Supplementary file 1 — Additional file 1 Figure S1. Relations of structural properties with ordered-mutation-inducing dynamics in BA network. A total of 250 BA random networks with |V| = 50 and |A| = 100 were generated. The time gap (T) was set to 1–10. (a) Mutation-sensitivity result with respect to the shortest path length. All pairs of nodes involving an FBL were classified into ‘Shorter-path direction’ and ‘Longer-path direction’ groups according that l(vi, vj) < l(vj, vi) and l(vi, vj) > l(vj, vi), respectively. (b) Mutation-sensitivity result with respect to the number of paths. All pairs of nodes were classified into ‘More-paths direction’ and ‘Fewer-paths direction’ groups according that n(vi, vj) > n(vj, vi) and n(vi, vj) < n(vj, vi), respectively. (c) Mutation-sensitivity result with respect to the FBLs. All pairs of nodes were classified into ‘FBL’ and ‘Non-FBL’ groups, according that any gene of the pair is involved by an FBL or not. (d) Order-specificity result with respect to the FBLs. All P-values were computed using the Mann-Whitney U test. Table S1. Gene information of HCS consisting 1192 genes, including its association with drug-target, tumor suppressor, and oncogene. Table S2. Gene information of KEGG consisting 1659 genes, including its association with drug-target, tumor suppressor, and oncogene. Table S3. Gene information of TGL consisting 61 genes, including its association with drug-target, tumor suppressor, and oncogene. [file 12920_2019_651_MOESM1_ESM.pdf]

Effects of ordered mutations on dynamics in signaling networks

Maulida Mazaya[1], Hung-Cuong Trinh[2], and Yung-Keun Kwon[1]*

[1]School of IT Convergence, University of Ulsan, 93 Daehak-ro, Nam-gu, Ulsan 44610, Republic of Korea.

[2]Faculty of Information Technology, Ton Duc Thank University, Ho Chi Minh City, Vietnam.

* Corresponding author

E-mail: maulidamazaya@gmail.com (MM), trinhhungcuong@tdtu.edu.vn (THC), kwonyk@ulsan.ac.kr (YKK)

Supporting information

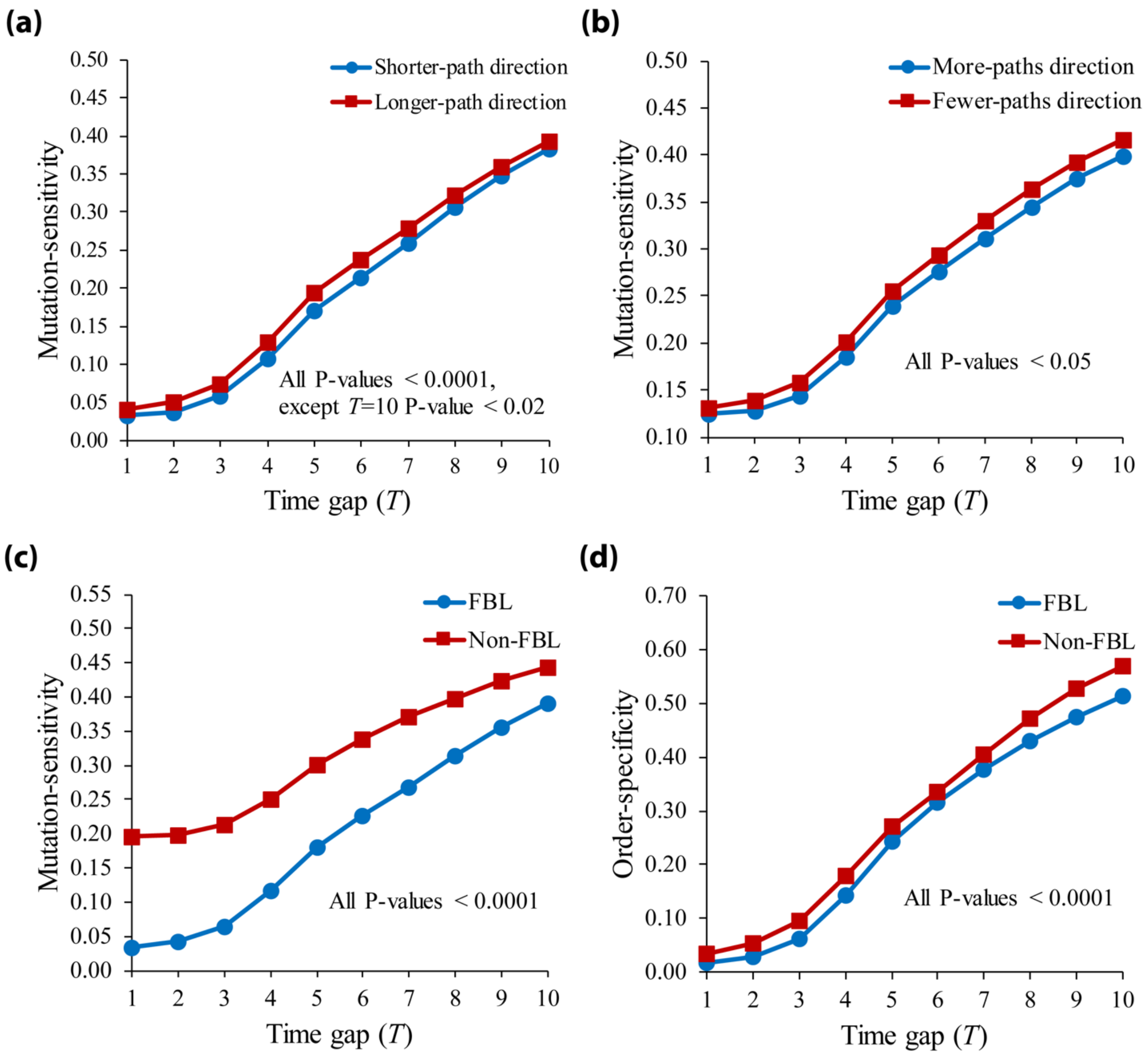

Fig. S1. Relations of structural properties with ordered-mutation-inducing dynamics in BA network. A total of 250 BA random networks with $|V| = 50$ and $|A| = 100$ were generated. The time gap (T) was set to 1-10. **(a)** Mutation-sensitivity result with respect to the shortest path length. All pairs of nodes involving an FBL were classified into 'Shorter-path direction' and 'Longer-path direction' groups according that $l(v_i, v_j) < l(v_j, v_i)$ and $l(v_i, v_j) > l(v_j, v_i)$, respectively. **(b)** Mutation-sensitivity result with respect to the number of paths. All pairs of nodes were classified into 'More-paths direction' and 'Fewer-paths direction' groups according that $n(v_i, v_j) > n(v_j, v_i)$ and $n(v_i, v_j) < n(v_j, v_i)$, respectively. **(c)** Mutation-sensitivity result with respect to the FBLs. All pairs of nodes were classified into 'FBL' and 'Non-FBL' groups, according that any gene of the pair is involved by an FBL or not. **(d)** Order-specificity result with respect to the FBLs. All P-values were computed using the Mann-Whitney U test.

Table S1. Gene information of HCS.

Gene name	Drug-target (1: True, 0: False)	Drug ID (-: unknown)	Tumor suppressor (1: True, 0: False)	Oncogene (1: True, 0: False)
SHARP	0	-	0	0
STAT1	0	-	1	0
RIN1	0	-	0	0
HAT1	0	-	0	0
CD14	0	-	0	0
PP2C	0	-	0	0
DYNAMIN	0	-	0	0
ETS1	0	-	0	1
GR	1	DB03147	0	0
SRF	0	-	0	0
PKAc	0	-	0	0
EIF4G	0	-	0	0
NTF5	1	DB00806	0	0
MUNC18	0	-	0	0
Calpastatin	1	DB01373	0	0
SMAD2	1	DB04522	1	0
CALM3	1	DB11093	0	0
AC1	1	DB00131	0	0
DAXX	0	-	0	1
ATF1	1	DB00852	0	1
Calpain2	0	-	0	0
HUS1	0	-	0	0
ENKEPHALIN	0	-	0	0
SEK	0	-	0	0
PTP	0	-	0	0
PAC1	0	-	1	1
SAM68	0	-	0	0
P15	0	-	0	0
ER	0	-	1	0
EPAC1	0	-	0	0
GJA1	1	DB01136	1	0
ARF1	1	DB02774	1	0
RIM	0	-	0	0
IKBB	0	-	0	0
FGFR	1	DB00039	0	0
GAIP	0	-	0	0
TIP1	0	-	0	0
RALB	0	-	0	0
PLSCR1	0	-	0	0
IFNy	1	DB01250	0	0
VDAC2	1	DB01375	0	0

cAMPGEFII	0	-	0	0
B2AR	1	DB00182	0	0
GNA12	0	-	0	1
E3	1	DB02717	0	0
CDC25C	0	-	0	1
INPP5A	0	-	0	0
PRAK	0	-	0	0
Ub	0	-	0	0
IL12RB2	0	-	0	0
CypA	1	DB00091	0	0
PAR2	0	-	1	0
HDAC	1	DB07553	0	0
CCT4	0	-	0	0
SYNAPTOTAGMIN	0	-	0	0
CAMK2A	1	DB04119	0	0
Ser-prot	0	-	0	0
PDK2	1	DB08608	0	0
PDGFR	1	DB00102	0	1
HIPK2	1	DB12010	1	0
JAG2	0	-	0	0
FIVEHT1CR	1	DB00193	0	0
HBX	0	-	0	0
I-FLICE	0	-	0	1
Dopamine	0	-	0	0
ARHGAP5	0	-	0	0
ADDUCIN	0	-	0	0
ARHGAP6	0	-	0	0
MITR	1	DB00313	0	0
EIF3S2	0	-	0	1
EGR2	0	-	1	0
PSD95	0	-	0	0
ANF	0	-	0	0
ETS2	0	-	1	1
ACTIN	0	-	0	0
CTCF	0	-	1	0
Clk	1	DB04367	0	0
MRLC	0	-	0	0
EIF4F	0	-	0	1
RANBP2	0	-	0	0
GAS2	0	-	0	0
c-JUN	1	DB01782	0	1
CYCS	1	DB01017	0	0
ICAT	0	-	1	0
ISRE	0	-	0	0

GLUT4	0	-	0	0
PTCH2	0	-	1	1
PAX2	0	-	0	1
PKG	0	-	0	0
cADPR	0	-	0	0
EIF4A	0	-	0	0
GPCR	0	-	0	0
ITGB4	1	DB05122	0	0
DJ1	1	DB09130	1	1
PPP3R2	1	DB00091	0	0
PTEN	1	DB04327	1	0
SV2A	1	DB01202	0	0
dATP	0	-	0	0
PLG	1	DB00009	0	0
TIF2	1	DB01645	0	0
MITF	0	-	0	1
TYK2	1	DB04716	0	0
pGC	0	-	0	0
DLC2	0	-	1	0
CB2R	1	DB00470	0	0
AMISYN	0	-	0	0
ACK	1	DB00171	0	0
PTPN12	0	-	1	0
D2R	1	DB00182	0	0
IRS2	0	-	0	1
MNK1	0	-	0	0
GDP	0	-	0	0
DNAPK	0	-	0	0
RIIa	0	-	0	0
ZAK	1	DB01254	0	0
RSK2	0	-	0	0
CHREBP	0	-	0	0
UBE2D3	0	-	0	0
RIP	0	-	0	1
CEK1	0	-	0	0
FAP	0	-	0	0
IFNAR	1	DB00008	0	0
SEROTONIN	0	-	0	0
Rho	1	DB01159	0	1
CDK9	1	DB03496	0	0
DAG	0	-	0	0
TP53BP1	0	-	1	0
FOS	1	DB08813	0	1
CTNND1	0	-	1	0

mDIA	0	-	0	0
CDC25	0	-	0	1
LTYPECA	0	-	0	0
WNT6	0	-	0	0
Ryk	0	-	0	0
KIR41	0	-	0	0
ROC1	0	-	0	1
IP3	0	-	0	0
Grb7	0	-	0	0
MAP1B	0	-	0	0
IGF1R	1	DB00030	0	1
CASP3	1	DB01017	0	0
IC261	0	-	0	0
E1A	0	-	0	0
TOR	0	-	0	0
PPARA	1	DB00132	1	0
SP3	0	-	0	0
PI3Ky	1	DB02010	0	0
NBS1	0	-	1	0
IAP	0	-	0	0
NO	0	-	0	0
AKAP13	0	-	0	1
SHANK	0	-	0	0
PIP3	0	-	0	0
SLC9A1	1	DB00594	0	0
DP1	0	-	0	0
MUNC13	0	-	0	0
TGIF	0	-	0	0
GRB2	1	DB00061	0	0
ARTS	0	-	1	0
GAB	0	-	0	0
IRAK-M	0	-	0	0
RLC	0	-	0	0
MIP1B	0	-	0	0
AMPK	1	DB00945	1	0
LMNA	0	-	0	0
p190RhoGAP	0	-	0	0
GRIT	0	-	0	0
LEF1	1	DB00903	0	1
SNAPIN	0	-	0	0
CD45	0	-	1	0
PFN1	1	DB07908	1	0
M2R	1	DB00202	0	0
Ca++	0	-	0	0

CK1e	1	DB00163	0	0
HAX1	0	-	0	1
GSK3A	1	DB12010	0	1
TRADD	0	-	0	0
CXCR4	1	DB00452	0	1
MEF2C	0	-	0	1
SPAL	0	-	0	0
NOTCH2	0	-	1	0
RAP30	0	-	0	0
p67phox	0	-	0	0
MUSK	1	DB12010	0	0
SMPD1	1	DB00381	0	0
ADAM17	1	DB06943	0	0
AXIN	1	DB04447	1	0
RGS4	0	-	0	0
Calpain1	1	DB04276	0	0
SSTR2	1	DB00104	0	0
BRCA1	0	-	1	0
RB	1	DB00030	1	0
FIBRONECTIN	0	-	0	0
AA	0	-	0	0
SnoN	0	-	0	1
PKLR	1	DB00119	0	0
Puma	0	-	1	0
AChE	1	DB00122	1	0
GATA4	0	-	1	0
ARHGEF11	0	-	0	0
PQCaCh	0	-	0	0
SHC	1	DB02139	0	0
Gbg	1	DB02459	0	0
Bmf	0	-	1	0
MMP7	1	DB00786	0	0
SOD	1	DB03382	0	0
DREAM	0	-	0	0
I1	0	-	0	0
DRE	0	-	0	0
SHIP	0	-	0	0
GABABR	1	DB00181	0	0
MAP2	1	DB01196	0	0
M3/6	0	-	0	0
NUMB	0	-	1	0
BNIP3L	0	-	1	0
ATF2	1	DB00852	0	0
ENDOPHILIN	0	-	0	0

CBL	0	-	1	1
Cathepsin	1	DB02140	0	0
CEBPA	0	-	1	0
GALPHAZ	0	-	0	0
IL6	1	DB01404	0	0
RGS2	0	-	0	0
SMURF1	0	-	0	1
TBR1	0	-	0	0
p90RSK	0	-	0	0
cIAP2	0	-	0	1
DARPP-32	0	-	1	0
GAB2	0	-	0	1
PDE3A	1	DB00235	0	0
BCL2	1	DB01050	0	1
F3	1	DB00036	0	0
EIF2B	0	-	0	0
MKP2	0	-	0	0
BLNK	0	-	1	0
D1R	1	DB00246	0	0
DOC2	0	-	1	0
TFIIB	0	-	0	0
P50	0	-	1	0
VPAC2R	0	-	0	0
GA	0	-	1	0
CAMP	1	DB02345	0	0
Lgs	0	-	1	1
Cby	0	-	0	0
INSR	1	DB00030	0	0
CK2	1	DB01765	0	0
MLC	0	-	0	0
EPHB2	1	DB04395	1	0
IKKy	0	-	0	0
DAAM1	0	-	0	0
ALK3	0	-	1	0
SPECTRIN	0	-	0	0
CORTACTIN	0	-	0	0
IL6R	1	DB06273	0	0
bRAF	1	DB00398	0	1
AKT	1	DB07812	0	1
RASGRP3	0	-	0	0
CKI	1	DB08325	0	0
CDC34	0	-	0	0
PCAF	1	DB01992	0	0
HBEGF	0	-	0	0

ITGB1	1	DB00098	1	0
KV12	1	DB06637	0	0
MAP4K3	1	DB12010	0	0
CSF1R	1	DB00619	0	1
TOB	0	-	0	0
ARF	0	-	1	0
PLK3	1	DB12010	0	0
Dynein	0	-	0	0
MGLUR7	1	DB00142	0	0
IKKe	0	-	0	1
VRK1	0	-	0	0
SHIP2	0	-	0	1
CCND1	1	DB01169	0	1
AMPAR	1	DB00898	0	0
POLR1B	0	-	0	0
CSNK1E	1	DB06195	0	0
JAK3	1	DB04716	0	0
Sin3	0	-	0	0
HDAC5	1	DB05015	0	0
KV41	0	-	0	0
GCAP	1	DB00848	0	0
FYN	1	DB01254	0	1
TRE	0	-	0	1
H2O2	0	-	0	0
CRY	1	DB03147	0	0
PKI	1	DB03374	0	0
SPRED2	0	-	0	0
TLR2	1	DB00045	0	0
GADD45	0	-	1	0
UBE3A	0	-	0	0
GNA16	0	-	0	0
HBO1	0	-	0	0
NMT	1	DB02180	0	0
AKAP	0	-	0	0
GAP	1	DB02263	0	0
PLC	1	DB02225	0	0
SNAP25	1	DB00083	0	0
MFNG	0	-	0	1
PPP2R2A	1	DB02506	0	0
SMAD7	0	-	0	0
RHEB	1	DB04137	0	0
GLYT1	1	DB00145	0	0
E2	1	DB04330	0	0
MKK7	0	-	0	0

PTPA	1	DB07813	1	0
TEC	1	DB12010	1	1
BIRC5	1	DB00206	0	1
NCS1	1	DB11093	0	0
NFM	0	-	0	0
HIPPOCALCIN	0	-	0	0
GRP1	0	-	0	0
CTNNB1	1	DB03904	0	1
REQ	0	-	0	0
CSK	1	DB01254	0	0
FODRIN	0	-	0	0
TCF7	0	-	0	0
WAVE2	0	-	0	0
PDE4B	1	DB00131	0	0
PTMA	0	-	0	1
M4R	1	DB00246	0	0
CAP	0	-	0	1
ARFGAP	0	-	0	0
GROUCHO	0	-	0	0
DISHEVELED	0	-	0	0
Gat2	0	-	0	0
SS	0	-	0	0
CLOCK	0	-	0	0
KRT18	0	-	0	0
CEM15	0	-	0	0
DIA	0	-	0	0
CHOP10	0	-	0	1
SRE	0	-	0	0
SOS1	0	-	0	0
HMGN1	0	-	0	0
ANKYRIN	0	-	0	0
TRYPSIN	1	DB03127	0	0
TNKS	0	-	0	0
PRK2	0	-	0	0
LARG	0	-	1	0
PDGF	0	-	0	0
EF1A	0	-	1	1
p120	0	-	1	0
IGF1	1	DB01890	1	0
SUMO1	0	-	0	0
AHR	1	DB00338	1	0
AR	1	DB00255	0	1
PDE3B	1	DB01640	0	0
P73	0	-	1	1

SKP1	1	DB01750	0	0
BMPR2	1	DB11639	1	0
7TMR	0	-	0	0
PTPB1	1	DB06989	0	0
PIP2	0	-	0	0
FRS2	0	-	0	0
MGLUR1	1	DB00142	0	1
RGS	0	-	0	1
EEF2K	0	-	0	0
eIF2B	0	-	0	0
p25	0	-	0	1
ADAM12	0	-	0	0
NFYA	0	-	0	0
RET	1	DB00398	0	1
IL1R	1	DB00026	0	0
TSA	1	DB02153	0	0
S1P	0	-	0	0
DFFB	0	-	0	0
RALGDS	0	-	0	1
STAT6	0	-	0	0
PDE	0	-	0	0
bARK	1	DB00171	0	0
C3G	0	-	0	0
CTIP	0	-	1	0
CLATHRIN	0	-	0	0
CPI17	0	-	0	1
HDAC3	1	DB02546	1	0
COLLAGEN	1	DB00048	0	0
LZK	0	-	0	0
FURIN	1	DB03600	0	0
NFH	0	-	0	0
BIM	0	-	1	0
CASP1	1	DB01017	0	0
AC2	1	DB02587	0	0
PDE6G	1	DB00203	0	0
STAMBPL1	0	-	0	0
ICAD	0	-	1	0
TNF	1	DB00005	0	0
AFX	0	-	1	0
FZD2	0	-	0	1
EPOR	1	DB00012	0	0
Hsp40	0	-	1	0
NGFR	0	-	1	0
AC	0	-	0	0

SHP2	1	DB02779	1	1
ARHGEF5	0	-	0	1
SNX6	0	-	0	0
GABAAR	1	DB08848	0	0
KOPR	1	DB00193	0	0
FRAT1	0	-	0	1
VILIP	0	-	0	0
RhoGTP	0	-	0	0
CAMKPASE	0	-	0	0
p32	0	-	0	0
UBE1L	0	-	0	0
SKIP	0	-	0	0
PP32	0	-	1	0
D3R	1	DB00246	0	0
DOCK180	0	-	0	0
G1	0	-	0	0
VASP	0	-	0	0
CABIN1	0	-	0	1
KIT	1	DB00398	0	1
CCND2	0	-	0	0
BETAARRESTIN	0	-	0	0
GFR	0	-	0	0
NIK	0	-	0	0
NAKED	0	-	0	0
NOPR	1	DB01497	0	0
ITGA1	0	-	0	0
PDE1C	1	DB00201	0	0
TRAF6	0	-	0	1
ERBIN	0	-	0	0
MLK3	0	-	0	0
TBK1	1	DB12010	0	0
ARHGEF6	0	-	0	0
ABL1	1	DB00171	0	1
CALMODULIN	1	DB00477	0	0
CALCINEURIN	0	-	0	0
PLCb	0	-	0	0
CYP19A1	1	DB00184	0	0
PAR4	0	-	1	0
SMRT	0	-	0	0
DHPG	0	-	0	0
ROS	0	-	0	1
WNT2	0	-	0	1
IL1B	1	DB01017	0	0
PHKA2	0	-	0	0

REA	0	-	0	0
Aven	0	-	0	0
Fibrinogen	1	DB00009	0	0
SOS2	0	-	0	0
PKN	0	-	0	0
DG	0	-	0	0
MKP1	0	-	0	0
GATA1	0	-	0	1
CTBP2	0	-	0	1
MAPKAP2	1	DB00945	0	0
SMAD3	0	-	0	0
cPLA2	0	-	1	0
VAV	0	-	0	1
LRP6	0	-	0	0
ROCK2	1	DB08162	0	0
PMCA	0	-	0	0
GAB1	0	-	0	1
SORCIN	0	-	0	0
HIF1A	1	DB01136	1	0
IL2	1	DB00852	0	0
IL4R	1	DB05078	0	0
XDH	1	DB00437	0	0
D4-GDI	0	-	0	0
PSEN1	0	-	0	0
EGFR	1	DB00002	0	1
TRKB	1	DB00321	0	0
EFA6	0	-	0	0
EGF	1	DB00364	0	0
BI-1	0	-	0	0
ENG	0	-	0	0
THC	1	DB00825	0	0
CCNE1	0	-	0	1
ADAM10	1	DB04991	0	0
PDE1B	1	DB00622	0	0
AMP	1	DB00131	0	0
EEF2	1	DB02059	0	0
RABGDI	0	-	0	0
G2	0	-	0	0
NFkB	1	DB00945	0	0
MOPR	1	DB00193	0	0
SNAI1	0	-	0	1
EBP	1	DB00675	0	0
MBP	0	-	0	0
DKK	0	-	0	0

IBP	0	-	0	0
RIP140	1	DB06884	0	0
ZAP70	1	DB02010	0	0
P35	0	-	0	0
CASK	1	DB01942	0	0
TGFBR2	1	DB09462	1	0
TCF3	0	-	1	1
XIAP	1	DB02628	0	1
SLP-76	0	-	0	0
NGF	1	DB01407	0	0
ITM2B	0	-	0	0
GAS	0	-	0	0
WNT	0	-	0	0
FAS	0	-	1	1
EIF3	0	-	0	0
FOUREBP1	0	-	0	0
ARHGEF1	0	-	0	1
IL1A	1	DB06372	0	0
SMO	1	DB01047	0	1
BAD	1	DB12340	0	0
AXAM2	0	-	0	0
FRIZZLED	0	-	0	0
Akt	1	DB07812	0	1
Forskolin	0	-	0	0
Mos	0	-	0	1
GEF	0	-	0	1
NICD	0	-	0	0
CAD	1	DB00128	0	1
CPI	0	-	0	0
AC5	1	DB02587	0	0
LMNB1	0	-	0	0
FNTA	1	DB04893	0	0
PGC1A	0	-	0	0
TAMALIN	0	-	0	0
CDH1	0	-	1	1
PYGB	1	DB00114	0	0
MAP1A	1	DB01196	0	0
CSNK1D	0	-	0	0
PIAS	0	-	0	0
GLYCINE	0	-	0	0
dsRNA	0	-	0	0
Sara	0	-	0	0
LMNB2	0	-	0	0
APOER2	0	-	0	0

ILPIP	0	-	0	0
PIAS4	0	-	0	0
SDF1	1	DB06822	0	0
hsp70	0	-	0	1
GIRK	1	DB00898	0	0
DD	1	DB00485	0	0
MAPK11	1	DB05157	0	0
SAP102	0	-	0	0
UXT	0	-	0	0
SMAD5	0	-	0	0
RASGAP	0	-	0	0
RAS	0	-	0	1
MEKK2	0	-	0	0
RAB3	0	-	0	0
TIEG2	0	-	0	0
BCL2L1	1	DB07108	0	1
ATP	0	-	0	0
SAP97	0	-	1	0
FRP	1	DB03247	1	0
FBI1	0	-	0	1
ZEB1	0	-	0	0
GLI1	0	-	1	1
CYTOCHROMEC	1	DB01017	0	0
GLI2	0	-	0	1
SPHK1	0	-	0	0
Hrk	0	-	0	0
MKP4	0	-	1	0
DOPR	1	DB00246	0	0
XAF1	0	-	1	0
CAPRI	0	-	0	0
DSH	0	-	0	0
NOTCH4	0	-	0	1
IRAK4	1	DB08590	0	0
LCK	1	DB01254	0	1
TSC1	0	-	1	0
Bcl-b	0	-	0	0
FILAMIN	0	-	0	0
HOP	0	-	1	0
BR1R	0	-	0	0
ErbB3	0	-	0	1
ROCK1	1	DB04707	0	1
ITGA6	0	-	0	0
BMAL1	0	-	1	0
CD44	1	DB06550	1	0

PKCE	0	-	1	1
ACC	1	DB00121	0	0
MAPK9	1	DB07020	1	0
NCK	0	-	0	0
ARP23	1	DB08235	0	0
MEKK4	0	-	1	0
GCK	1	DB02379	0	0
BRADYKININ	0	-	0	0
CL	0	-	0	0
NLK	0	-	0	0
SYNTROPHIN	0	-	0	0
Livin	0	-	0	0
DGK	0	-	0	0
SHP1	0	-	1	0
ALPHAACTININ	0	-	0	0
PAK1	1	DB12010	0	1
CAMKK	0	-	0	0
AIF	1	DB03147	0	0
PDGFRA	1	DB00102	0	1
DOK	0	-	0	0
MEK1	1	DB02152	0	0
FANCD2	0	-	0	0
CCR5	1	DB04835	0	0
MAP4K6	0	-	0	0
POR1	0	-	0	0
EPHRIN	0	-	0	0
P1433	0	-	0	0
Cd++	0	-	0	0
RPS6	1	DB11638	0	0
CD4	1	DB00098	1	0
BTK	1	DB01254	1	0
VAMP	0	-	0	0
STAT2	0	-	0	0
DELTA	1	DB00675	0	1
MIG1	0	-	0	0
IQGAP	0	-	0	0
Gai	0	-	0	0
LEF	1	DB01883	0	0
BAG1	1	DB07045	0	0
TCF	1	DB04419	1	0
STAT	0	-	0	0
SODD	0	-	0	0
Tpl-2	0	-	1	1
Ab-R	0	-	0	0

RAP1GAP	0	-	1	0
HSPG	1	DB00020	0	0
LIMK1	1	DB08912	0	0
RKIP	0	-	1	0
NAP	0	-	0	0
ECSIT	0	-	0	0
DcR3	0	-	0	0
Nucling	0	-	0	0
DAB1	0	-	0	0
O2	0	-	0	0
PLASMIN	0	-	0	0
DEDD1	0	-	0	0
SPRED1	0	-	0	0
SYNGAP	0	-	0	0
PDE5A	1	DB00203	0	0
CREM	0	-	1	0
p85	0	-	0	1
ARP3	1	DB08235	0	0
IRSP53	0	-	0	0
MCL1	1	DB09401	0	1
HINT1	1	DB00131	1	0
WIF1	0	-	1	0
ADAPTIN	0	-	0	0
BPAG2	0	-	0	0
GDNF	1	DB09301	0	0
Era	1	DB04022	1	0
IBR	0	-	0	0
EEF1A2	0	-	0	1
ARNIP	0	-	1	0
MAP3K2	1	DB06616	0	0
NTRK1	1	DB00321	0	1
CTBP1	1	DB01942	0	0
TAB2	0	-	0	0
IKBA	0	-	0	0
RIC	0	-	0	0
TSAP6	0	-	0	0
MYOSIN	0	-	0	0
CREB	1	DB00131	0	1
CEACAM1	1	DB00113	1	0
PI3P	0	-	0	0
cAR	1	DB01708	1	0
CDK1	1	DB02052	0	1
SIAH	0	-	0	0
PDK1	1	DB07403	0	0

SYNTAXIN	0	-	0	0
CRK	0	-	0	1
RASGRP1	0	-	0	1
PIB5PA	0	-	0	0
FIVEHT1AR	1	DB00216	0	0
CryAB	0	-	0	1
Graf	0	-	0	0
A1R	1	DB00201	0	0
SF1	1	DB11638	0	0
Glycogen	1	DB00114	0	0
PKA	1	DB01919	0	0
CDK5	1	DB02052	0	0
MAP-1	0	-	0	0
F13	0	-	0	0
EIF-4EBP	0	-	0	0
FKBP	0	-	0	0
CHK2	1	DB05149	1	0
p53	1	DB00945	1	0
CASP10	0	-	0	0
PDE2	1	DB00201	0	0
PP2B	0	-	0	0
STAT5	0	-	1	0
RACK	0	-	0	0
TGFB	1	DB00070	0	1
BID	0	-	0	0
p38gamma	1	DB02482	0	0
SH3KBP1	0	-	0	0
RAP1	0	-	1	1
GRB10	0	-	0	0
ARNO	1	DB01863	0	0
ANAPC1	0	-	1	0
ERK2	1	DB02010	0	0
SODIUM	0	-	0	0
IL13RA2	1	DB05078	0	0
CALNEXIN	1	DB00025	0	0
TGFBR3	0	-	1	0
MEF2D	0	-	0	0
GABA	0	-	0	0
GAQ	0	-	0	1
MAL	1	DB00114	1	0
GKAP	0	-	0	0
MYOSINV	0	-	0	0
DLK	0	-	1	0
LPL	1	DB06439	0	0

IKKA	0	-	0	0
AVP	0	-	0	0
SIVA1	1	DB01593	0	0
IL8	1	DB05434	0	0
TRIO	0	-	0	1
PDPK1	1	DB00482	0	0
FADD	0	-	1	0
MAP4K5	1	DB12010	0	0
GALPHAO	0	-	0	0
CHLORIDE	0	-	0	0
MEK5	0	-	0	0
RAIDD	0	-	0	0
JNKK1	0	-	1	0
PLD	1	DB00122	0	0
ARHGAP4	0	-	0	0
Gas	0	-	0	0
THRAP2	0	-	0	0
GALPHAI	0	-	0	0
CK1d	1	DB03619	0	0
CHORDIN	0	-	0	0
APS	0	-	0	0
CRAC	0	-	0	0
PKCz	0	-	0	0
CASP2	0	-	1	0
ZNF259	0	-	0	0
NTYPECA	1	DB06616	0	0
CASP7	1	DB03384	0	0
MLK1	1	DB08703	0	0
ERK1	1	DB01064	0	0
CARD14	0	-	0	0
N-WASP	0	-	0	0
MST1R	1	DB12010	1	1
PABP	0	-	0	0
NEDD8	0	-	0	0
CDK2	1	DB01888	1	0
H3	0	-	0	0
TSC2	0	-	1	0
BAP31	0	-	0	0
JAK2	1	DB04716	0	1
Bok	0	-	0	0
RAL	0	-	0	0
PCNA	1	DB00279	0	0
CERAMIDE	0	-	0	0
A20	0	-	1	0

SRC	1	DB01254	0	1
p110Ia	0	-	0	0
GAP43	0	-	0	0
RIN	0	-	0	0
NUR77	0	-	1	0
L1	1	DB03958	0	0
HDAC1	1	DB01169	1	1
GSK3B	1	DB01356	1	0
ELK1	0	-	0	1
PTP-SL	0	-	0	0
Miz1	0	-	0	0
CAMK2	1	DB07168	0	0
SAG	0	-	0	1
NEUROFIBROMIN	0	-	0	0
FANCF	0	-	0	0
VIL2	0	-	0	0
RAF1	1	DB00398	0	1
SKP2	0	-	1	1
SPINOPHILIN	0	-	0	0
TAB1	1	DB06757	0	0
RAD51	1	DB04395	0	0
TFF1	1	DB00481	0	0
PERK	0	-	0	0
PIM2	0	-	0	1
MTOR	1	DB00337	0	1
TERT	1	DB00495	0	0
RAP74	0	-	0	0
SYK	1	DB02010	1	1
APOLLON	0	-	0	0
ERK5	1	DB06616	0	0
PLCg-2	0	-	0	0
TBX2	0	-	0	1
DR3	0	-	0	0
EIF3S1	0	-	0	0
CARM1	0	-	0	0
NMDAR	1	DB00142	0	0
CDK6	1	DB03496	1	1
RELN	0	-	0	0
CITRON	0	-	0	0
CHN1	0	-	0	0
BCL10	0	-	1	0
FASCIN	0	-	0	0
MP1	0	-	0	0
PER	0	-	0	0

TNFR2	1	DB00005	0	1
KV11	1	DB00228	0	0
RELA	1	DB02836	0	0
BFAR	0	-	0	0
CRMP2	0	-	0	0
DAB2	0	-	1	0
BAK	1	DB01050	0	0
BCR	1	DB01254	1	1
ARFGEF	0	-	0	0
GCN2	0	-	0	0
MSK1	0	-	0	0
ATR	0	-	1	0
CHK1	1	DB05149	1	0
SMARCA4	0	-	1	0
NAIP	0	-	0	0
ASC	0	-	1	0
PRF1	0	-	0	0
PKC	0	-	0	0
DAPK	1	DB04069	1	0
PDE1A	1	DB00622	0	0
RAD9	0	-	0	0
POTASSIUM	0	-	0	0
IL2RG	1	DB00004	0	0
nPKC	1	DB00675	0	0
PARP	1	DB02498	1	0
COFILIN1	1	DB04147	0	0
RADIXIN	0	-	0	0
FIVEHT2AR	1	DB00246	0	0
CD28	0	-	0	0
RABPHILIN	0	-	0	0
MAPK13	1	DB05157	0	0
PLA2P	0	-	0	0
CTLA-4	1	DB06186	0	0
CDK4	1	DB02733	0	1
IRF3	0	-	1	0
SENP1	0	-	0	0
ACH	1	DB00184	0	1
LEP	0	-	0	0
p16	0	-	1	0
ARP2	1	DB08235	0	0
COPS5	0	-	0	1
STAT5B	1	DB01254	0	0
GTP	0	-	0	0
TAK1	0	-	1	1

PRKCD	1	DB04376	1	0
F2	1	DB00001	0	0
PIP5K	0	-	0	0
MKP5	0	-	0	0
RHOGEFs	0	-	0	0
SSTR1	1	DB00104	0	0
Cn	0	-	0	0
CK19	0	-	1	0
PAK2	1	DB12010	0	0
CGMP	0	-	0	0
cIAP	0	-	0	0
PI3K	1	DB00201	0	1
GNA13	0	-	0	1
CASP9	0	-	0	0
DYRK	1	DB07608	0	0
INHIBITOR2	1	DB05341	0	0
ANANDAMIDE	0	-	0	0
PROFILIN	0	-	0	0
HOMER	0	-	0	0
IKK2	1	DB00244	0	0
cIAP1	0	-	0	1
IL13RA1	0	-	0	0
P13K	1	DB01064	0	0
KV42	1	DB00157	0	0
Gy	1	DB02127	0	0
SYNTAPHILIN	0	-	0	0
MAPKAP-K3	1	DB07728	0	0
MYD88	0	-	0	1
FLASH	0	-	0	0
ASAP1	0	-	0	0
PELP1	0	-	0	1
STAT4	0	-	0	0
PTC	0	-	0	1
RICS	0	-	0	0
POLR2A	0	-	0	0
ELK4	0	-	0	0
p68	0	-	0	0
MLCP	0	-	0	0
CB1R	1	DB00470	0	0
MNK2	0	-	0	0
GLI3	0	-	0	1
Importin	0	-	0	0
Cer	0	-	0	0
TCF4	0	-	1	0

p14ARF	0	-	1	0
PTK	1	DB07248	0	1
KIR21	0	-	0	0
ISGF3G	0	-	0	0
c-Myc	0	-	0	1
PKR	0	-	0	0
PRMT1	1	DB01752	0	0
TIAM1	0	-	0	0
Omi	0	-	1	0
Bcl-rambo	0	-	0	0
MEK6	0	-	0	0
ALPHA7NACHR	1	DB00184	0	0
GBETAGAMMA	0	-	0	0
HUR	0	-	0	1
RAN	1	DB04315	0	0
PSMC4	0	-	0	0
Hypoxia	0	-	0	0
MEKK	0	-	0	0
P21	0	-	1	1
CAVEOLIN	0	-	0	0
NRG	0	-	0	0
DAX-1	1	DB00755	0	0
IL-13	0	-	0	0
JNK3	1	DB01782	1	0
NOTCH3	0	-	1	0
FER	1	DB12010	0	0
PPP2CA	1	DB00163	1	0
PI-4-P5K	0	-	0	0
CALBRAIN	0	-	0	0
HSP27	0	-	0	1
PLCy	0	-	0	0
IRAK	0	-	0	0
CK1a	0	-	1	0
SUFU	0	-	1	0
RHOA	1	DB04315	1	1
GIT1	0	-	0	0
PKCA	1	DB00144	0	1
PR-A	0	-	0	0
CBP	1	DB08655	1	1
PLK1	1	DB06897	1	1
MADD	0	-	1	0
APP	1	DB00746	0	0
GHR	1	DB00052	0	0
PrP	1	DB00759	0	0

FASLG	0	-	0	0
ERb	1	DB08916	1	0
IP3R	0	-	0	0
STEP	0	-	0	0
A-Raf	1	DB00171	0	1
PA	0	-	0	0
MYT1	0	-	0	0
TGFBR1	1	DB03921	0	0
VITRONECTIN	1	DB00054	0	0
GALPHAS	0	-	0	0
p55gamma	1	DB04315	0	0
SOCS	0	-	0	0
p38	1	DB01761	1	1
CCND3	0	-	0	0
KIR23	0	-	0	0
WAVE3	0	-	0	0
FAP-1	0	-	1	0
EDNRA	1	DB00559	0	0
PROKR1	0	-	0	0
PDE6A	1	DB00201	0	0
JIP	1	DB02235	1	0
RYR	1	DB00201	0	0
CBLB	0	-	0	1
ECM	0	-	0	0
p300	0	-	0	0
PTP1B	1	DB01133	1	0
RSK	1	DB00945	1	0
RASGRF	0	-	0	0
LFNG	0	-	0	0
RANBP9	0	-	1	0
RGS16	0	-	0	0
DAP10	0	-	0	0
BPAG1	0	-	0	0
p65	0	-	0	1
PCLy	0	-	0	0
UBE2D2	1	DB02418	0	0
MSK2	0	-	0	0
ERT	0	-	0	0
CCNB1	0	-	0	1
BDNF	1	DB05047	0	0
PPP2R5C	1	DB02506	1	0
SERPINC1	1	DB00407	0	0
P35611	0	-	0	0
AGTR1	1	DB00177	1	0

NDUFS1	1	DB00157	0	0
ADENOSINE	0	-	0	0
TUBB4Q	1	DB05147	0	0
ALPHA2AR	1	DB00182	0	0
Ty	0	-	0	0
PAR1	1	DB00086	0	0
CCNA2	1	DB02091	0	0
PDE4A	1	DB00277	0	0
EIF5	0	-	0	0
PLD1	1	DB00122	1	0
CD47	0	-	0	0
HSP72	1	DB02424	0	1
A2AR	1	DB00201	0	0
Noxa	0	-	0	0
Idax	0	-	1	0
UBE2D1	0	-	0	0
E2F1	0	-	1	1
MET	1	DB02152	0	1
POFUT1	0	-	0	0
Itk	1	DB02010	0	1
NOS3	1	DB00125	0	0
FKHRL1	0	-	1	0
D1	0	-	0	0
COFILIN2	0	-	0	0
PAF	0	-	0	1
CASP6	0	-	0	0
LYN	1	DB01254	0	0
IFN-yR	1	DB00033	0	0
RNPK	0	-	0	0
PDE4D	1	DB00131	0	0
VEGFR	0	-	0	0
Hsp90	1	DB05036	0	0
EIF4B	0	-	0	0
K+	0	-	0	0
DLL1	0	-	0	0
MARCKS	0	-	1	0
DVL2	0	-	0	0
CAMKIV	1	DB07664	0	0
GZMB	1	DB01017	0	0
FGF	1	DB00686	0	0
CD3	1	DB00075	0	0
GRIP1	0	-	0	0
IRS1	1	DB08513	0	0
MLK	0	-	0	0

MYB	0	-	0	1
p85beta	1	DB01064	0	0
FAN	0	-	0	0
NCOR	0	-	0	0
PICK1	0	-	0	0
D2	0	-	0	0
RasGRP	0	-	0	1
Nrdp1	0	-	0	0
IL4	1	DB06560	0	0
PAFR	0	-	0	0
SH2D3C	0	-	0	0
JAG1	0	-	0	0
WAVE1	0	-	0	0
Ins	1	DB01593	0	0
PRKCG	1	DB00675	0	0
SMAD4	0	-	1	0
myosin	0	-	0	0
APLP1	1	DB01593	0	0
CAMK1	1	DB12010	0	0
I-mf	0	-	0	0
DJ2	0	-	0	0
RNTRE	0	-	0	0
GLYR	1	DB00145	0	0
PP2A	0	-	1	0
FANCE	0	-	0	0
PP1	0	-	0	0
MST3	0	-	0	0
MEF2B	0	-	0	0
PPP1CC	1	DB02169	0	0
BNIP3	0	-	0	0
RPTK	1	DB00072	0	0
BRCA2	0	-	1	0
ACTG	0	-	0	0
TNFR1	1	DB03507	0	0
TALIN	0	-	0	0
TFIID	0	-	0	0
EPS15	0	-	0	0
RAR	1	DB00210	0	1
KAR	1	DB00123	0	0
RAD17	0	-	0	0
CDK7	1	DB02482	0	0
GAT1	1	DB00906	0	0
GELSOLIN	1	DB02621	0	0
RhoGDP	0	-	0	0

RAP1A	0	-	1	1
CASP8	1	DB12651	1	0
RAP1B	0	-	0	0
ARNT	0	-	0	0
NFAT	0	-	0	0
APIP	0	-	0	0
LAMB1	1	DB06245	0	0
GLUTAMATE	0	-	0	0
MLCK	0	-	0	0
ID2	0	-	0	1
RAP2	1	DB04137	0	0
LAT	1	DB08071	0	0
TFF2	1	DB03088	0	0
PXN	0	-	0	0
ATM	1	DB00201	1	0
MAP4K1	1	DB12010	1	0
MKP3	0	-	1	0
GH	0	-	0	0
NOS1	1	DB00155	0	0
GC2	1	DB00142	0	0
ARHGAP1	0	-	0	0
CIR	1	DB00898	0	0
RTYPECA	0	-	0	0
DEFCAP	0	-	0	0
NOX1	1	DB09140	0	0
CD19	1	DB06342	0	0
RAC1	1	DB00993	0	1
CK2A2	1	DB07546	0	1
BTRC	0	-	0	0
TUBULIN	1	DB00518	0	0
BIK	0	-	1	0
CRE	0	-	0	0
ACHRE	1	DB00674	0	0
PP5	0	-	1	0
LEPR	1	DB05098	0	0
ASK1	0	-	0	0
EIF4E	1	DB01649	0	1
APAF1	1	DB00171	1	0
JAK1	1	DB02375	0	1
RALBP1	0	-	0	0
CRKL	0	-	0	1
MEKK3	0	-	0	0
MCF2	0	-	0	1
CAV2	0	-	0	0

MOR	1	DB03793	0	0
PAK6	1	DB12010	0	0
Catalase	1	DB01213	0	0
STAT3	1	DB05959	1	1
SMPD2	0	-	0	0
BAX	0	-	1	1
EGR1	0	-	1	0
TRAF2	0	-	1	0
PLCE	1	DB01103	1	0
BCLG	0	-	0	0
Estrogen	0	-	0	0
TAU	0	-	0	0
P130Cas	0	-	0	0
CLTC	0	-	0	0
DCP1A	0	-	0	0
SHP	1	DB03929	1	0
ALPHA1AR	1	DB00182	0	0
MEK2	0	-	0	0
KV14	0	-	0	0
FIVEHT4R	1	DB00604	0	0
Fe	0	-	0	0
Acinus	0	-	0	0
ACTN1	1	DB06773	0	0
Bcl-w	0	-	0	0
PIN1	1	DB01766	1	0
HCK	1	DB01809	0	0
SNCA	1	DB02709	0	0
JNK	1	DB01782	0	0
EPS8	0	-	0	1
CSP	0	-	0	0
N41	0	-	0	0
MURR1	0	-	0	0
TLR4	1	DB01183	0	0
M1R	1	DB00185	0	0
Gia	0	-	0	0
NE	1	DB00157	0	0
HO	0	-	0	0
14-3-3	0	-	0	1
AIP4	0	-	0	0
PDZGEF	0	-	0	0
SYNAPSIN	0	-	0	0
CITED1	0	-	0	0
Insulin	0	-	0	0
CAM	0	-	1	0

PDE4C	1	DB00651	0	0
FORKHEAD	0	-	0	0
APC	1	DB00055	1	0
LRP	0	-	0	0
VLDLR	1	DB03017	0	0
HRI	0	-	0	0
ERBB2	1	DB00072	0	1
AGTR2	1	DB01349	0	0
TWIST	0	-	0	1
Pygo	0	-	0	0
a2-Antiplasmin	0	-	0	0
MEF2	0	-	0	0
p107	0	-	1	0
SRA1	0	-	0	0
FAK	1	DB07248	0	0
ID3	0	-	0	0
p70s6K	0	-	0	0
Tb	0	-	0	0
CDC42	1	DB02623	0	0
Gb	1	DB03297	0	0
ILK	0	-	1	1
ADPRIBSYCYCLASE	0	-	0	0
KSR	0	-	0	0
XAP2	0	-	1	0
IFNA	0	-	0	0
VEGF	1	DB00112	1	0
TID1	0	-	1	0
GFAP	0	-	0	0
VAV2	0	-	0	1
SPRY	0	-	0	0
MDM2	1	DB01593	0	1
HIST3H3	0	-	0	0
PLA2	1	DB00795	1	0
PKD	0	-	0	0
Ku70	0	-	0	0
TUBBY	0	-	0	0
MBD3	0	-	0	0
CNG	0	-	0	0
SAC	0	-	0	0
PLCD1	1	DB03401	1	0
P2	1	DB04137	0	0
CALRETICULIN	1	DB00025	0	0
MEK3	0	-	0	0
GC1	1	DB00142	1	0

S6	0	-	0	0
p27	0	-	0	0
UBC9	0	-	0	0
EIF2A	0	-	0	0
SP1	0	-	0	0
CDC25A	0	-	0	1
CD36	0	-	0	0

Table S2. Gene information of KEGG.

Entrez Gene ID	Gene name	Drug-target (1: True, 0: False)	Drug ID (-: unknown)	Tumor suppressor (1: True, 0: False)	Oncogene (1: True, 0: False)
387	RHOA	1	DB04315	1	1
2885	GRB2	1	DB00061	0	0
2549	GAB1	0	-	0	1
25	ABL1	1	DB00171	0	1
6093	ROCK1	1	DB04707	0	1
9475	ROCK2	1	DB08162	0	0
1398	CRK	0	-	0	1
1399	CRKL	0	-	0	1
5879	RAC1	1	DB00993	0	1
5880	RAC2	1	DB00514	0	0
5881	RAC3	0	-	0	1
6714	SRC	1	DB01254	0	1
3667	IRS1	1	DB08513	0	0
8471	IRS4	0	-	0	0
8660	IRS2	0	-	0	1
7294	TXK	1	DB12010	0	0
5499	PPP1CA	1	DB02506	1	0
5500	PPP1CB	0	-	0	0
5501	PPP1CC	1	DB02169	0	0
2911	GRM1	1	DB00142	0	1
2915	GRM5	1	DB00659	0	0
2932	GSK3B	1	DB01356	1	0
5058	PAK1	1	DB12010	0	1
2776	GNAQ	0	-	0	1
1956	EGFR	1	DB00002	0	1
9020	MAP3K14	0	-	0	0
836	CASP3	1	DB01017	0	0
2889	RAPGEF1	0	-	0	0
2775	GNAO1	0	-	0	0
867	CBL	0	-	1	1
868	CBLB	0	-	0	1
23624	CBLC	0	-	0	0
2149	F2R	1	DB00086	0	0
998	CDC42	1	DB02623	0	0
5601	MAPK9	1	DB07020	1	0
5599	MAPK8	1	DB01782	0	0
5602	MAPK10	1	DB01782	1	0
4659	PPP1R12A	0	-	0	0
5335	PLCG1	0	-	0	0
2534	FYN	1	DB01254	0	1
4067	LYN	1	DB01254	0	0

815	CAMK2A	1	DB04119	0	0
816	CAMK2B	1	DB07168	0	0
817	CAMK2D	1	DB07853	0	0
818	CAMK2G	1	DB06616	0	0
5900	RALGDS	0	-	0	1
5502	PPP1R1A	0	-	0	0
5781	PTPN11	1	DB02779	1	1
1630	DCC	0	-	1	0
5336	PLCG2	0	-	0	0
23365	ARHGEF12	0	-	1	0
7409	VAV1	0	-	0	1
7410	VAV2	0	-	0	1
10451	VAV3	0	-	0	1
2768	GNA12	0	-	0	1
5747	PTK2	1	DB07248	0	0
1855	DVL1	0	-	0	0
1856	DVL2	0	-	0	0
1857	DVL3	0	-	0	0
3265	HRAS	1	DB02210	0	1
3845	KRAS	1	DB07771	0	1
4893	NRAS	0	-	0	1
7048	TGFBR2	1	DB09462	1	0
5290	PIK3CA	1	DB00171	0	1
5291	PIK3CB	1	DB00201	0	0
5293	PIK3CD	1	DB00201	0	0
5294	PIK3CG	1	DB02010	0	0
5906	RAP1A	0	-	1	1
5908	RAP1B	0	-	0	0
1499	CTNNB1	1	DB03904	0	1
5295	PIK3R1	1	DB01064	0	1
5296	PIK3R2	1	DB01064	0	0
8503	PIK3R3	1	DB01064	0	0
23533	PIK3R5	0	-	0	0
2147	F2	1	DB00001	0	0
3688	ITGB1	1	DB00098	1	0
9138	ARHGEF1	0	-	0	1
8772	FADD	0	-	1	0
10672	GNA13	0	-	0	1
27040	LAT	1	DB08071	0	0
2475	MTOR	1	DB00337	0	1
1445	CSK	1	DB01254	0	0
1147	CHUK	1	DB00233	1	0
3551	IKBKB	1	DB00244	0	0
8517	IKBKG	1	DB04998	0	0

3937	LCP2	0	-	0	0
5590	PRKCZ	1	DB00675	0	0
5777	PTPN6	0	-	1	0
5579	PRKCB	1	DB00163	1	0
3480	IGF1R	1	DB00030	0	1
5340	PLG	1	DB00009	0	0
8826	IQGAP1	1	DB11638	0	0
5605	MAP2K2	1	DB06616	0	0
6654	SOS1	0	-	0	0
6655	SOS2	0	-	0	0
9846	GAB2	0	-	0	1
2890	GRIA1	1	DB00142	0	0
2891	GRIA2	1	DB00142	0	0
5578	PRKCA	1	DB00144	0	1
5582	PRKCG	1	DB00675	0	0
6009	RHEB	1	DB04137	0	0
5922	RASA2	0	-	0	0
7535	ZAP70	1	DB02010	0	0
2770	GNAI1	1	DB04315	0	0
2771	GNAI2	0	-	0	1
2773	GNAI3	0	-	0	0
9459	ARHGEF6	0	-	0	0
841	CASP8	1	DB12651	1	0
6850	SYK	1	DB02010	1	1
843	CASP10	0	-	0	0
5594	MAPK1	1	DB01064	0	0
5595	MAPK3	1	DB00605	0	0
25791	NGEF	0	-	0	0
2774	GNAL	0	-	0	0
55970	GNG12	0	-	0	0
7249	TSC2	0	-	1	0
3683	ITGAL	1	DB00095	0	0
3689	ITGB2	1	DB00641	0	0
1500	CTNND1	0	-	1	0
801	CALM1	1	DB00477	0	0
805	CALM2	1	DB11093	0	0
808	CALM3	1	DB11093	0	0
810	CALML3	0	-	0	0
51806	CALML5	0	-	0	0
163688	CALML6	0	-	0	0
8936	WASF1	0	-	0	0
10163	WASF2	0	-	0	0
2159	F10	1	DB00025	0	0
2207	FCER1G	1	DB00895	0	0

2066	ERBB4	1	DB08916	1	1
51744	CD244	0	-	0	0
3690	ITGB3	1	DB00054	1	0
3691	ITGB4	1	DB05122	0	0
3693	ITGB5	0	-	0	0
3694	ITGB6	0	-	0	0
3695	ITGB7	1	DB05122	0	0
3696	ITGB8	0	-	0	0
5159	PDGFRB	1	DB00102	0	1
2778	GNAS	1	DB02587	0	1
7186	TRAF2	0	-	1	0
8737	RIPK1	1	DB12010	0	0
1387	CREBBP	1	DB08655	1	0
2033	EP300	0	-	0	0
7040	TGFB1	1	DB00070	1	1
7042	TGFB2	0	-	0	0
7043	TGFB3	1	DB03316	0	0
5156	PDGFRA	1	DB00102	0	1
8440	NCK2	0	-	0	0
5921	RASA1	0	-	0	0
5062	PAK2	1	DB12010	0	0
4690	NCK1	0	-	0	0
6464	SHC1	0	-	0	0
25759	SHC2	0	-	0	0
53358	SHC3	0	-	0	0
399694	SHC4	0	-	0	0
5515	PPP2CA	1	DB00163	1	0
5516	PPP2CB	1	DB00163	1	0
5518	PPP2R1A	1	DB02506	0	1
5519	PPP2R1B	0	-	1	0
842	CASP9	0	-	0	0
6237	RRAS	0	-	0	1
22800	RRAS2	0	-	0	1
22808	MRAS	0	-	0	1
4615	MYD88	0	-	0	1
7157	TP53	1	DB00945	1	0
1839	HBEGF	0	-	0	0
1729	DIAPH1	0	-	0	0
1730	DIAPH2	0	-	0	0
23396	PIP5K1C	0	-	0	0
3676	ITGA4	1	DB00108	0	0
5170	PDPK1	1	DB00482	0	0
4638	MYLK	1	DB12010	0	0
85366	MYLK2	1	DB04825	0	0

91807	MYLK3	1	DB12010	0	0
5063	PAK3	1	DB12010	0	0
10298	PAK4	1	DB12010	0	0
56924	PAK6	1	DB12010	0	0
57144	PAK5	1	DB12010	0	1
27289	RND1	0	-	0	0
148022	TICAM1	0	-	0	0
3791	KDR	1	DB00398	0	0
369	ARAF	1	DB03142	0	1
4851	NOTCH1	0	-	1	1
4853	NOTCH2	0	-	1	0
4854	NOTCH3	0	-	1	0
4855	NOTCH4	0	-	0	1
5728	PTEN	1	DB04327	1	0
10842	PPP1R17	0	-	0	0
4233	MET	1	DB02152	0	1
5604	MAP2K1	1	DB02152	0	0
1793	DOCK1	0	-	0	0
10928	RALBP1	0	-	0	0
5530	PPP3CA	1	DB08231	0	0
5532	PPP3CB	0	-	0	0
5533	PPP3CC	0	-	1	0
5534	PPP3R1	1	DB08231	0	0
5535	PPP3R2	1	DB00091	0	0
11261	CHP1	1	DB11093	0	0
63928	CHP2	0	-	0	0
6091	ROBO1	0	-	1	0
940	CD28	0	-	0	0
50649	ARHGEF4	0	-	0	0
5364	PLXNB1	0	-	0	1
5365	PLXNB3	0	-	0	0
23654	PLXNB2	0	-	0	0
9855	FARP2	0	-	0	0
7132	TNFRSF1A	1	DB03507	0	0
7305	TYROBP	0	-	0	0
10746	MAP3K2	1	DB06616	0	0
10458	BAIAP2	0	-	0	0
3684	ITGAM	0	-	0	0
9901	SRGAP3	0	-	1	0
23380	SRGAP2	0	-	1	0
57522	SRGAP1	0	-	0	0
3643	INSR	1	DB00030	0	0
5898	RALA	1	DB04315	0	0
5899	RALB	0	-	0	0

3984	LIMK1	1	DB08912	0	0
3985	LIMK2	1	DB12010	0	0
572	BAD	1	DB12340	0	0
8717	TRADD	0	-	0	0
3383	ICAM1	1	DB00108	0	0
5609	MAP2K7	0	-	0	0
5894	RAF1	1	DB00398	0	1
4193	MDM2	1	DB01593	0	1
5624	PROC	1	DB00170	0	0
5361	PLXNA1	0	-	0	0
5362	PLXNA2	0	-	0	0
55558	PLXNA3	0	-	0	0
2260	FGFR1	1	DB00039	0	1
4089	SMAD4	0	-	1	0
595	CCND1	1	DB01169	0	1
894	CCND2	0	-	0	0
896	CCND3	0	-	0	0
2158	F9	1	DB00025	0	0
3554	IL1R1	1	DB00026	0	0
3680	ITGA9	0	-	0	0
355	FAS	0	-	1	1
3479	IGF1	1	DB01890	1	0
2161	F12	1	DB01593	0	0
3655	ITGA6	0	-	0	0
3672	ITGA1	0	-	0	0
3673	ITGA2	0	-	0	0
3674	ITGA2B	1	DB00054	0	0
3675	ITGA3	0	-	0	1
3678	ITGA5	1	DB02709	1	0
3679	ITGA7	0	-	1	0
3685	ITGAV	1	DB00098	1	0
8515	ITGA10	0	-	0	0
8516	ITGA8	0	-	0	0
22801	ITGA11	0	-	0	0
2322	FLT3	1	DB00398	1	1
6092	ROBO2	0	-	0	0
29851	ICOS	0	-	0	0
3815	KIT	1	DB00398	0	1
7852	CXCR4	1	DB00452	0	1
1910	EDNRB	1	DB00559	1	0
1394	CRHR1	1	DB09067	0	0
2065	ERBB3	0	-	0	1
2263	FGFR2	1	DB00039	0	1
2064	ERBB2	1	DB00072	0	1

7850	IL1R2	0	-	0	0
4301	AFDN	1	-	0	0
3932	LCK	1	DB01254	0	1
3802	KIR2DL1	0	-	0	0
3803	KIR2DL2	0	-	0	0
3804	KIR2DL3	0	-	0	0
3805	KIR2DL4	0	-	0	0
3811	KIR3DL1	0	-	0	0
3812	KIR3DL2	0	-	0	0
3821	KLRC1	0	-	0	0
3824	KLRD1	0	-	0	0
57292	KIR2DL5A	0	-	0	0
2767	GNA11	0	-	0	0
2769	GNA15	0	-	0	0
9630	GNA14	0	-	0	0
2781	GNAZ	0	-	0	0
1020	CDK5	1	DB02052	0	0
919	CD247	1	DB00075	0	0
4214	MAP3K1	1	DB06061	0	0
4052	LTBP1	0	-	0	0
4087	SMAD2	1	DB04522	1	0
4088	SMAD3	0	-	0	0
5328	PLAU	1	DB00013	0	0
7454	WAS	1	DB01731	0	0
8976	WASL	0	-	0	0
5327	PLAT	1	DB00013	0	0
1978	EIF4EBP1	0	-	0	0
394	ARHGAP5	0	-	0	0
2909	ARHGAP35	0	-	0	0
9564	BCAR1	0	-	0	0
353376	TICAM2	0	-	0	0
2535	FZD2	0	-	0	1
7855	FZD5	0	-	0	0
7976	FZD3	0	-	0	0
8321	FZD1	0	-	0	0
8322	FZD4	0	-	0	0
8323	FZD6	0	-	0	0
8324	FZD7	0	-	0	0
8325	FZD8	0	-	0	0
8326	FZD9	0	-	0	0
11211	FZD10	0	-	0	0
814	CAMK4	1	DB07664	0	0
5566	PRKACA	1	DB01919	0	1
5567	PRKACB	1	DB02482	0	0

5568	PRKACG	0	-	0	0
5613	PRKX	0	-	0	0
5616	PRKY	0	-	0	0
1969	EPHA2	1	DB01254	1	1
2041	EPHA1	1	DB12010	1	0
2042	EPHA3	1	DB12010	1	0
2043	EPHA4	1	DB12010	0	0
2044	EPHA5	1	DB01254	0	0
2045	EPHA7	1	DB07970	0	0
2046	EPHA8	1	DB12010	0	0
285220	EPHA6	1	DB12010	0	0
2798	GNRHR	1	DB00007	0	0
56288	PARD3	0	-	0	0
5255	PHKA1	0	-	0	0
5256	PHKA2	0	-	0	0
5257	PHKB	0	-	0	0
5260	PHKG1	1	DB12010	0	0
5261	PHKG2	0	-	0	0
2245	FGD1	0	-	0	0
89846	FGD3	0	-	0	0
4068	SH2D1A	0	-	0	0
28964	GIT1	0	-	0	0
83593	RASSF5	0	-	1	0
10810	WASF3	0	-	0	0
3973	LHCGR	1	DB00014	0	0
3624	INHBA	0	-	0	0
3625	INHBB	0	-	0	0
3626	INHBC	0	-	0	0
83729	INHBE	0	-	0	0
3071	NCKAP1L	0	-	0	0
10152	ABI2	0	-	1	0
10787	NCKAP1	0	-	0	0
23191	CYFIP1	0	-	0	0
26999	CYFIP2	0	-	0	0
8793	TNFRSF10D	0	-	0	0
8794	TNFRSF10C	0	-	0	0
8795	TNFRSF10B	1	DB05895	1	0
8797	TNFRSF10A	0	-	1	0
207	AKT1	1	DB00171	0	1
208	AKT2	1	DB07812	0	1
10000	AKT3	0	-	0	0
2185	PTK2B	1	DB01097	0	1
7133	TNFRSF1B	1	DB00005	0	1
3556	IL1RAP	0	-	0	0

7056	THBD	1	DB00055	1	0
2321	FLT1	1	DB00398	0	0
2324	FLT4	1	DB00398	0	0
695	BTK	1	DB01254	1	0
1027	CDKN1B	0	-	1	1
1385	CREB1	1	DB00131	0	1
3645	INSRR	1	DB12010	0	0
468	ATF4	1	DB00852	0	0
9586	CREB5	0	-	0	0
10488	CREB3	0	-	0	0
64764	CREB3L2	0	-	0	0
84699	CREB3L3	0	-	0	0
90993	CREB3L1	0	-	1	0
148327	CREB3L4	0	-	0	0
5054	SERPINE1	1	DB00009	0	0
81624	DIAPH3	0	-	0	0
4157	MC1R	1	DB04931	0	0
1947	EFNB1	0	-	0	0
1948	EFNB2	0	-	0	0
1949	EFNB3	0	-	0	0
1026	CDKN1A	1	DB01169	1	1
4633	MYL2	0	-	0	0
4636	MYL5	0	-	0	0
10398	MYL9	0	-	0	0
10627	MYL12A	1	DB08378	0	0
29895	MYLPF	0	-	0	0
58498	MYL7	0	-	0	0
93408	MYL10	0	-	0	0
103910	MYL12B	0	-	0	0
7189	TRAF6	0	-	0	1
7099	TLR4	1	DB01183	0	0
5350	PLN	0	-	0	0
1616	DAXX	0	-	0	1
346562	GNAT3	0	-	0	0
2261	FGFR3	1	DB00039	0	1
5111	PCNA	1	DB00279	0	0
5584	PRKCI	1	DB03777	0	1
1017	CDK2	1	DB01888	1	0
55845	BRK1	0	-	0	0
2264	FGFR4	1	DB00039	0	1
1028	CDKN1C	0	-	1	0
91	ACVR1B	1	DB00171	0	0
983	CDK1	1	DB02052	0	1
7187	TRAF3	0	-	0	0

890	CCNA2	1	DB02091	0	0
8900	CCNA1	0	-	0	0
7471	WNT1	0	-	0	1
7472	WNT2	0	-	0	1
7473	WNT3	0	-	0	1
7474	WNT5A	0	-	1	1
7475	WNT6	0	-	0	0
7476	WNT7A	0	-	1	0
7477	WNT7B	0	-	0	0
7478	WNT8A	0	-	0	0
7479	WNT8B	0	-	0	0
7480	WNT10B	0	-	0	1
7481	WNT11	0	-	1	0
7482	WNT2B	0	-	0	0
7483	WNT9A	0	-	0	0
7484	WNT9B	0	-	0	0
51384	WNT16	0	-	0	0
54361	WNT4	0	-	0	0
80326	WNT10A	0	-	0	1
81029	WNT5B	0	-	0	0
89780	WNT3A	0	-	0	0
840	CASP7	1	DB03384	0	0
3654	IRAK1	1	DB12010	0	0
1019	CDK4	1	DB02733	0	1
1021	CDK6	1	DB03496	1	1
4216	MAP3K4	1	DB12010	1	0
1030	CDKN2B	0	-	1	0
7046	TGFBR1	1	DB03921	0	0
130399	ACVR1C	0	-	1	0
2241	FER	1	DB12010	0	0
1487	CTBP1	1	DB01942	0	0
1488	CTBP2	0	-	0	1
650	BMP2	0	-	1	0
4838	NODAL	0	-	0	0
51135	IRAK4	1	DB08590	0	0
8554	PIAS1	0	-	1	0
9063	PIAS2	0	-	0	0
9641	IKBKE	1	DB12010	0	1
10401	PIAS3	0	-	0	0
29110	TBK1	1	DB12010	0	0
51588	PIAS4	0	-	0	0
655	BMP7	0	-	0	1
8200	GDF5	1	DB02325	0	0
898	CCNE1	0	-	0	1

9134	CCNE2	0	-	0	0
596	BCL2	1	DB01050	0	1
9088	PKMYT1	1	DB12010	0	0
10092	ARPC5	1	DB08235	0	0
81873	ARPC5L	0	-	0	0
268	AMH	0	-	1	0
959	CD40LG	1	DB06475	0	0
4086	SMAD1	0	-	0	0
4090	SMAD5	0	-	0	0
4093	SMAD9	0	-	0	0
5627	PROS1	1	DB00055	0	0
114609	TIRAP	0	-	0	0
941	CD80	1	DB01281	0	0
3065	HDAC1	1	DB01169	1	1
3066	HDAC2	1	DB00227	0	0
3818	KLKB1	1	DB01593	0	0
3827	KNG1	1	DB01593	0	0
3384	ICAM2	0	-	0	0
3385	ICAM3	0	-	0	0
652	BMP4	1	DB01373	1	0
653	BMP5	0	-	0	0
654	BMP6	0	-	0	0
656	BMP8B	0	-	0	0
151449	GDF7	0	-	0	0
353500	BMP8A	0	-	0	0
392255	GDF6	0	-	0	0
4991	OR1D2	0	-	0	0
4992	OR1F1	0	-	0	0
4993	OR2C1	0	-	0	0
4994	OR3A1	0	-	0	0
4995	OR3A2	0	-	0	0
7932	OR2H2	0	-	0	0
8383	OR1A1	0	-	0	0
8385	-	0	-	0	0
8386	OR1D5	0	-	0	0
8387	OR1E1	0	-	0	0
8388	OR1E2	0	-	0	0
8390	OR1G1	0	-	0	0
8392	OR3A3	0	-	0	0
8590	OR6A2	0	-	0	0
10798	OR5I1	0	-	0	0
23538	OR52A1	0	-	0	0
26188	OR1C1	0	-	0	0
26189	OR1A2	0	-	0	0

26211	OR2F1	0	-	0	0
26212	OR2B6	0	-	0	0
26219	OR1J4	0	-	0	0
26245	OR2M4	0	-	0	0
26246	OR2L2	0	-	0	0
26248	OR2K2	0	-	0	0
26333	OR7A17	0	-	0	0
26338	OR5L2	0	-	0	0
26339	OR5K1	0	-	0	0
26476	OR10J1	0	-	0	0
26492	OR8G2P	0	-	0	0
26493	OR8B8	0	-	0	0
26494	OR8G1	0	-	0	0
26496	OR10A3	0	-	0	0
26529	OR12D2	0	-	0	0
26531	OR11A1	0	-	0	0
26532	OR10H3	0	-	0	0
26533	OR10G3	0	-	0	0
26534	OR10G2	0	-	0	0
26538	OR10H2	0	-	0	0
26539	OR10H1	0	-	0	0
26658	OR7C2	0	-	0	0
26659	OR7A5	0	-	0	0
26664	OR7C1	0	-	0	0
26682	OR4F4	0	-	0	0
26683	OR4F3	0	-	0	0
26686	OR4E2	0	-	0	0
26689	OR4D1	0	-	0	0
26692	OR2W1	0	-	0	0
26696	OR2T1	0	-	0	0
26707	OR2J2	0	-	0	0
26716	OR2H1	0	-	0	0
26735	OR1L3	0	-	0	0
26737	OR1L1	0	-	0	0
26740	OR1J2	0	-	0	0
56656	OR2S2	0	-	0	0
79290	OR13A1	0	-	0	0
79295	OR5H6	0	-	0	0
79310	OR5H2	0	-	0	0
79317	OR4K5	0	-	0	0
79324	OR51G1	0	-	0	0
79339	OR51B4	0	-	0	0
79345	OR51B2	0	-	0	0
79473	OR52N1	0	-	0	0

79501	OR4F5	0	-	0	0
79541	OR2A4	0	-	0	0
79544	OR4K1	0	-	0	0
81050	OR5AC2	0	-	0	0
81061	OR11H1	0	-	0	0
81099	OR4F17	0	-	0	0
81127	OR4K15	0	-	0	0
81168	OR8J3	0	-	0	0
81282	OR51G2	0	-	0	0
81285	OR51E2	0	-	0	0
81300	OR4P4	0	-	0	0
81309	OR4C15	0	-	0	0
81318	OR4A5	0	-	0	0
81327	OR4A16	0	-	0	0
81328	OR4A15	0	-	0	0
81392	OR2AE1	0	-	0	0
81399	OR4F16	0	-	0	0
81442	OR6N2	0	-	0	0
81448	OR6K2	0	-	0	0
81469	OR2G3	0	-	0	0
81470	OR2G2	0	-	0	0
81472	OR2C3	0	-	0	0
81696	OR5V1	0	-	0	0
81697	OR2B2	0	-	0	0
81797	OR12D3	0	-	0	0
119678	OR52E2	0	-	0	0
119679	OR52J3	0	-	0	0
119682	OR51L1	0	-	0	0
119687	OR51A7	0	-	0	0
119692	OR51S1	0	-	0	0
119694	OR51F2	0	-	0	0
119695	OR52R1	0	-	0	0
119749	OR4C46	0	-	0	0
119764	OR4X2	0	-	0	0
119765	OR4B1	0	-	0	0
119772	OR52M1	0	-	0	0
119774	OR52K2	0	-	0	0
120065	OR5P2	0	-	0	0
120066	OR5P3	0	-	0	0
120586	OR8I2	0	-	0	0
120775	OR2D3	0	-	0	0
120776	OR2D2	0	-	0	0
120787	OR52W1	0	-	0	0
120793	OR56A4	0	-	0	0

120796	OR56A1	0	-	0	0
121130	OR10P1	0	-	0	0
121275	OR10AD1	0	-	0	0
121364	OR10A7	0	-	0	0
122740	OR4K14	0	-	0	0
122742	OR4L1	0	-	0	0
122748	OR11H6	0	-	0	0
124538	OR4D2	0	-	0	0
125958	OR7D4	0	-	0	0
125962	OR7G1	0	-	0	0
125963	OR1M1	0	-	0	0
126370	OR1I1	0	-	0	0
126541	OR10H4	0	-	0	0
127059	OR2M5	0	-	0	0
127062	OR2M3	0	-	0	0
127064	OR2T12	0	-	0	0
127066	OR14C36	0	-	0	0
127068	OR2T34	0	-	0	0
127069	OR2T10	0	-	0	0
127074	OR2T4	0	-	0	0
127077	OR2T11	0	-	0	0
127385	OR10J5	0	-	0	0
127623	OR2B11	0	-	0	0
128360	OR10T2	0	-	0	0
128367	OR10X1	0	-	0	0
128368	OR10Z1	0	-	0	0
128371	OR6K6	0	-	0	0
128372	OR6N1	0	-	0	0
130075	OR9A4	0	-	0	0
134083	OR2Y1	0	-	0	0
135924	OR9A2	0	-	0	0
135941	OR2A14	0	-	0	0
135946	OR6B1	0	-	0	0
135948	OR2F2	0	-	0	0
138799	OR13C5	0	-	0	0
138802	OR13C8	0	-	0	0
138803	OR13C3	0	-	0	0
138804	OR13C4	0	-	0	0
138805	OR13F1	0	-	0	0
138881	OR1L8	0	-	0	0
138882	OR1N2	0	-	0	0
138883	OR1N1	0	-	0	0
143496	OR52B4	0	-	0	0
143502	OR52I2	0	-	0	0

143503	OR51E1	0	-	0	0
144124	OR10A5	0	-	0	0
144125	OR2AG1	0	-	0	0
150681	OR6B3	0	-	0	0
158131	OR1Q1	0	-	0	0
162998	OR7D2	0	-	0	0
196335	OR56B4	0	-	0	0
219417	OR8U1	0	-	0	0
219428	OR4C16	0	-	0	0
219429	OR4C11	0	-	0	0
219431	OR4S2	0	-	0	0
219432	OR4C6	0	-	0	0
219436	OR5D14	0	-	0	0
219437	OR5L1	0	-	0	0
219438	OR5D18	0	-	0	0
219447	OR5AS1	0	-	0	0
219453	OR8K5	0	-	0	0
219464	OR5T2	0	-	0	0
219469	OR8H1	0	-	0	0
219473	OR8K3	0	-	0	0
219477	OR8J1	0	-	0	0
219479	OR5R1	0	-	0	0
219482	OR5M3	0	-	0	0
219484	OR5M8	0	-	0	0
219487	OR5M11	0	-	0	0
219493	OR5AR1	0	-	0	0
219858	OR8B12	0	-	0	0
219865	OR8G5	0	-	0	0
219869	OR10G8	0	-	0	0
219870	OR10G9	0	-	0	0
219873	OR10S1	0	-	0	0
219874	OR6T1	0	-	0	0
219875	OR4D5	0	-	0	0
219952	OR6Q1	0	-	0	0
219954	OR9I1	0	-	0	0
219956	OR9Q1	0	-	0	0
219957	OR9Q2	0	-	0	0
219958	OR1S2	0	-	0	0
219959	OR1S1	0	-	0	0
219960	OR10Q1	0	-	0	0
219965	OR5B17	0	-	0	0
219968	OR5B21	0	-	0	0
219981	OR5A2	0	-	0	0
219982	OR5A1	0	-	0	0

219983	OR4D6	0	-	0	0
219986	OR4D11	0	-	0	0
254783	OR6C74	0	-	0	0
254786	OR6C3	0	-	0	0
254879	OR2T6	0	-	0	0
254973	OR1L4	0	-	0	0
255725	OR52B2	0	-	0	0
256144	OR4C3	0	-	0	0
256148	OR4S1	0	-	0	0
256892	OR51F1	0	-	0	0
282763	OR51B5	0	-	0	0
282770	OR10AG1	0	-	0	0
282775	OR5J2	0	-	0	0
283092	OR4C13	0	-	0	0
283093	OR4C12	0	-	0	0
283111	OR51V1	0	-	0	0
283159	OR8D1	0	-	0	0
283160	OR8D2	0	-	0	0
283162	OR8B4	0	-	0	0
283189	OR9G4	0	-	0	0
283297	OR10A4	0	-	0	0
283365	OR6C6	0	-	0	0
283694	OR4N4	0	-	0	0
284383	OR2Z1	0	-	0	0
284433	OR10H5	0	-	0	0
284521	OR2L13	0	-	0	0
284532	OR14A16	0	-	0	0
285659	OR2V2	0	-	0	0
286362	OR13C9	0	-	0	0
286365	OR13D1	0	-	0	0
338662	OR8D4	0	-	0	0
338674	OR5F1	0	-	0	0
338675	OR5AP2	0	-	0	0
338751	OR52L1	0	-	0	0
338755	OR2AG2	0	-	0	0
340980	OR52B6	0	-	0	0
341152	OR2AT4	0	-	0	0
341276	OR10A2	0	-	0	0
341416	OR6C2	0	-	0	0
341418	OR6C4	0	-	0	0
341568	OR8S1	0	-	0	0
341799	OR6S1	0	-	0	0
343169	OR6F1	0	-	0	0
343171	OR2W3	0	-	0	0

343172	OR2T8	0	-	0	0
343173	OR2T3	0	-	0	0
343406	OR10R2	0	-	0	0
343563	OR2T29	0	-	0	0
346517	OR6V1	0	-	0	0
346525	OR2A12	0	-	0	0
346528	OR2A1	0	-	0	0
347168	OR1J1	0	-	0	0
347169	OR1B1	0	-	0	0
347468	OR13H1	0	-	0	0
387748	OR56B1	0	-	0	0
389090	OR6B2	0	-	0	0
390036	OR52K1	0	-	0	0
390037	OR52I1	0	-	0	0
390038	OR51D1	0	-	0	0
390054	OR52A5	0	-	0	0
390058	OR51B6	0	-	0	0
390059	OR51M1	0	-	0	0
390061	OR51Q1	0	-	0	0
390063	OR51I1	0	-	0	0
390064	OR51I2	0	-	0	0
390066	OR52D1	0	-	0	0
390067	OR52H1	0	-	0	0
390072	OR52N4	0	-	0	0
390075	OR52N5	0	-	0	0
390077	OR52N2	0	-	0	0
390078	OR52E6	0	-	0	0
390079	OR52E8	0	-	0	0
390081	OR52E4	0	-	0	0
390083	OR56A3	0	-	0	0
390084	OR56A5	0	-	0	0
390093	OR10A6	0	-	0	0
390113	OR4X1	0	-	0	0
390142	OR5D13	0	-	0	0
390144	OR5D16	0	-	0	0
390151	OR8H2	0	-	0	0
390152	OR8H3	0	-	0	0
390154	OR5T3	0	-	0	0
390155	OR5T1	0	-	0	0
390157	OR8K1	0	-	0	0
390162	OR5M9	0	-	0	0
390167	OR5M10	0	-	0	0
390168	OR5M1	0	-	0	0
390174	OR9G1	0	-	0	0

390181	OR5AK2	0	-	0	0
390191	OR5B12	0	-	0	0
390195	OR5AN1	0	-	0	0
390197	OR4D10	0	-	0	0
390199	OR4D9	0	-	0	0
390201	OR10V1	0	-	0	0
390260	OR6X1	0	-	0	0
390261	OR6M1	0	-	0	0
390264	OR10G4	0	-	0	0
390265	OR10G7	0	-	0	0
390275	OR8A1	0	-	0	0
390321	OR6C1	0	-	0	0
390323	OR6C75	0	-	0	0
390326	OR6C76	0	-	0	0
390327	OR6C70	0	-	0	0
390429	OR4N2	0	-	0	0
390431	OR4K2	0	-	0	0
390433	OR4K13	0	-	0	0
390436	OR4K17	0	-	0	0
390437	OR4N5	0	-	0	0
390439	OR11G2	0	-	0	0
390442	OR11H4	0	-	0	0
390445	OR5AU1	0	-	0	0
390538	OR4M2	0	-	0	0
390648	OR4F6	0	-	0	0
390649	OR4F15	0	-	0	0
390882	OR7G2	0	-	0	0
390883	OR7G3	0	-	0	0
390892	OR7A10	0	-	0	0
391107	OR10K2	0	-	0	0
391109	OR10K1	0	-	0	0
391112	OR6Y1	0	-	0	0
391114	OR6K3	0	-	0	0
391189	OR11L1	0	-	0	0
391190	OR2L8	0	-	0	0
391191	OR2AK2	0	-	0	0
391192	OR2L3	0	-	0	0
391194	OR2M2	0	-	0	0
391195	OR2T33	0	-	0	0
391196	OR2M7	0	-	0	0
391211	OR2G6	0	-	0	0
392138	OR2A25	0	-	0	0
392309	OR13J1	0	-	0	0
392376	OR13C2	0	-	0	0

392390	OR1L6	0	-	0	0
392391	OR5C1	0	-	0	0
392392	OR1K1	0	-	0	0
393046	OR2A5	0	-	0	0
401427	OR2A7	0	-	0	0
401665	OR51T1	0	-	0	0
401666	OR51A4	0	-	0	0
401667	OR51A2	0	-	0	0
401992	OR2T2	0	-	0	0
401993	OR2T5	0	-	0	0
401994	OR14I1	0	-	0	0
402135	OR5K2	0	-	0	0
402317	OR2A42	0	-	0	0
441608	OR5B3	0	-	0	0
441639	OR9K2	0	-	0	0
441669	OR4Q3	0	-	0	0
441670	OR4M1	0	-	0	0
441911	OR10J3	0	-	0	0
441933	OR13G1	0	-	0	0
442186	OR2J3	0	-	0	0
442191	OR14J1	0	-	0	0
442194	OR10C1	0	-	0	0
442361	OR2A2	0	-	0	0
504189	OR8U8	0	-	0	0
2	A2M	1	DB00102	0	0
27	ABL2	1	DB00171	0	1
31	ACACA	1	DB00121	0	0
32	ACACB	1	DB00121	0	0
52	ACP1	1	DB00173	0	0
58	ACTA1	1	DB02621	0	0
59	ACTA2	1	DB12695	0	0
60	ACTB	1	DB04216	0	0
70	ACTC1	0	-	0	0
71	ACTG1	1	DB09130	0	0
72	ACTG2	0	-	0	0
90	ACVR1	1	DB00171	0	0
92	ACVR2A	1	DB12118	0	0
93	ACVR2B	0	-	0	0
94	ACVRL1	1	DB00171	0	0
102	ADAM10	1	DB04991	0	0
107	ADCY1	1	DB00131	0	0
108	ADCY2	1	DB02587	0	0
109	ADCY3	0	-	0	0
111	ADCY5	1	DB02587	0	0

112	ADCY6	0	-	0	0
113	ADCY7	0	-	0	0
114	ADCY8	0	-	0	0
115	ADCY9	0	-	0	0
135	ADORA2A	1	DB00201	0	0
136	ADORA2B	1	DB00277	0	0
146	ADRA1D	1	DB00211	0	0
147	ADRA1B	1	DB00211	0	0
148	ADRA1A	1	DB00211	0	0
153	ADRB1	1	DB00127	0	0
154	ADRB2	1	DB00127	0	0
155	ADRB3	1	DB00368	0	0
157	GRK3	1	-	0	1
182	JAG1	0	-	0	0
185	AGTR1	1	DB00177	1	0
269	AMHR2	1	DB00171	0	0
317	APAF1	1	DB00171	1	0
324	APC	1	DB00055	1	0
329	BIRC2	0	-	0	1
330	BIRC3	0	-	0	1
331	XIAP	1	DB02628	0	1
356	FASLG	0	-	0	0
374	AREG	0	-	0	0
406	ARNTL	0	-	1	0
409	ARRB2	0	-	0	0
434	ASIP	0	-	0	0
462	SERPINC1	1	DB00407	0	0
487	ATP2A1	1	DB03909	0	0
488	ATP2A2	1	DB06157	0	0
489	ATP2A3	0	-	0	0
552	AVPR1A	1	DB00035	0	0
553	AVPR1B	1	DB00035	0	0
598	BCL2L1	1	DB07108	0	1
613	BCR	1	DB01254	1	1
623	BDKRB1	1	DB00178	0	0
624	BDKRB2	1	DB05038	0	0
637	BID	0	-	0	0
657	BMPR1A	1	DB11639	1	0
658	BMPR1B	1	DB12010	0	0
659	BMPR2	1	DB11639	1	0
673	BRAF	1	DB00398	0	1
685	BTC	0	-	0	0
839	CASP6	0	-	0	0
857	CAV1	0	-	1	1

858	CAV2	0	-	0	0
859	CAV3	0	-	0	0
861	RUNX1	0	-	1	1
862	RUNX1T1	0	-	0	1
886	CCKAR	1	DB00403	0	0
887	CCKBR	1	DB00183	0	0
891	CCNB1	0	-	0	1
925	CD8A	0	-	0	0
926	CD8B	0	-	0	0
929	CD14	0	-	0	0
930	CD19	1	DB06342	0	0
942	CD86	1	DB00098	0	0
948	CD36	0	-	0	0
958	CD40	1	DB06360	0	0
960	CD44	1	DB06550	1	0
961	CD47	0	-	0	0
962	CD48	0	-	0	0
990	CDC6	0	-	0	1
1022	CDK7	1	DB02482	0	0
1029	CDKN2A	0	-	1	0
1031	CDKN2C	0	-	1	0
1032	CDKN2D	0	-	0	0
1050	CEBPA	0	-	1	0
1072	CFL1	1	DB04147	0	0
1073	CFL2	0	-	0	0
1080	CFTR	1	DB00171	1	0
1081	CGA	1	-	0	0
1101	CHAD	0	-	0	0
1128	CHRM1	1	DB00185	0	0
1129	CHRM2	1	DB00202	0	0
1131	CHRM3	1	DB00185	0	0
1132	CHRM4	1	DB00246	0	0
1133	CHRM5	1	DB00246	0	0
1154	CISH	0	-	0	0
1270	CNTF	0	-	0	0
1271	CNTFR	0	-	0	0
1277	COL1A1	1	DB00048	0	0
1278	COL1A2	1	DB00048	0	0
1280	COL2A1	1	DB00048	0	0
1281	COL3A1	1	DB00048	0	0
1282	COL4A1	0	-	0	0
1284	COL4A2	0	-	0	0
1286	COL4A4	0	-	0	0
1288	COL4A6	0	-	0	0

1289	COL5A1	0	-	0	0
1290	COL5A2	0	-	0	0
1291	COL6A1	0	-	0	0
1292	COL6A2	0	-	0	0
1293	COL6A3	0	-	0	0
1301	COL11A1	0	-	0	0
1302	COL11A2	0	-	0	0
1311	COMP	1	DB01373	0	0
1392	CRH	1	DB01285	0	0
1407	CRY1	0	-	0	0
1408	CRY2	0	-	0	0
1432	MAPK14	1	DB01254	0	0
1437	CSF2	1	DB05194	1	0
1438	CSF2RA	1	DB00020	0	0
1439	CSF2RB	1	DB00020	0	0
1440	CSF3	0	-	0	1
1441	CSF3R	1	DB00019	0	1
1442	CSH1	0	-	0	0
1452	CSNK1A1	1	DB12010	1	0
1453	CSNK1D	0	-	0	0
1454	CSNK1E	1	DB06195	0	0
1455	CSNK1G2	1	DB03083	0	0
1456	CSNK1G3	1	DB04751	0	0
1457	CSNK2A1	1	DB00171	0	1
1459	CSNK2A2	1	DB07546	0	1
1460	CSNK2B	1	DB00171	0	0
1489	CTF1	0	-	0	0
1493	CTLA4	1	DB06186	0	0
1511	CTSG	1	DB02360	0	0
1535	CYBA	1	DB00514	0	0
1536	CYBB	1	DB00514	0	0
1605	DAG1	0	-	0	0
1634	DCN	1	DB03754	1	0
1647	GADD45A	0	-	1	0
1676	DFFA	0	-	1	0
1808	DPYSL2	1	DB11638	0	0
1812	DRD1	1	DB00246	0	0
1813	DRD2	1	DB00182	0	0
1816	DRD5	1	DB00246	0	0
1840	DTX1	0	-	0	0
1843	DUSP1	0	-	1	1
1844	DUSP2	0	-	0	0
1845	DUSP3	0	-	0	0
1846	DUSP4	0	-	0	0

1847	DUSP5	0	-	1	0
1848	DUSP6	0	-	1	0
1849	DUSP7	0	-	0	0
1850	DUSP8	0	-	0	0
1852	DUSP9	0	-	1	0
1869	E2F1	0	-	1	1
1870	E2F2	0	-	1	0
1871	E2F3	0	-	1	1
1874	E2F4	0	-	0	0
1875	E2F5	0	-	0	1
1902	LPAR1	0	-	0	0
1906	EDN1	1	DB05407	0	0
1909	EDNRA	1	DB00559	0	0
1942	EFNA1	0	-	0	0
1943	EFNA2	0	-	0	0
1944	EFNA3	0	-	0	0
1945	EFNA4	0	-	0	0
1946	EFNA5	0	-	1	0
1950	EGF	1	DB00364	0	0
1977	EIF4E	1	DB01649	0	1
2047	EPHB1	1	DB12010	0	0
2048	EPHB2	1	DB04395	1	0
2049	EPHB3	0	-	1	0
2050	EPHB4	1	DB01254	1	0
2051	EPHB6	1	DB12010	1	0
2056	EPO	0	-	0	0
2057	EPOR	1	DB00012	0	0
2069	EREG	0	-	0	0
2081	ERN1	1	DB07382	0	0
2113	ETS1	0	-	0	1
2122	MECOM	0	-	0	1
2150	F2RL1	0	-	0	0
2151	F2RL2	0	-	0	0
2152	F3	1	DB00036	0	0
2153	F5	1	DB00055	0	0
2155	F7	1	DB00036	0	0
2157	F8	1	DB00055	0	0
2160	F11	1	DB00100	0	0
2162	F13A1	1	DB01839	0	0
2165	F13B	1	DB01593	0	0
2206	MS4A2	1	DB00043	0	0
2242	FES	1	DB12010	0	1
2243	FGA	1	DB00009	0	0
2244	FGB	1	DB00364	0	0

2246	FGF1	1	DB00686	0	0
2247	FGF2	1	DB00364	0	0
2248	FGF3	0	-	0	1
2249	FGF4	1	DB00686	0	1
2250	FGF5	0	-	0	1
2251	FGF6	0	-	0	1
2252	FGF7	0	-	0	0
2253	FGF8	0	-	0	1
2254	FGF9	0	-	0	0
2255	FGF10	0	-	0	1
2256	FGF11	0	-	0	0
2257	FGF12	0	-	0	0
2258	FGF13	0	-	0	0
2259	FGF14	0	-	0	0
2266	FGG	1	DB00364	0	0
2277	VEGFD	0	-	0	0
2308	FOXO1	0	-	1	1
2309	FOXO3	0	-	1	0
2323	FLT3LG	0	-	0	0
2335	FN1	1	DB01593	0	0
2353	FOS	1	DB08813	0	1
2488	FSHB	0	-	0	0
2492	FSHR	1	DB00032	0	0
2648	KAT2A	1	DB01992	0	0
2688	GH1	0	-	0	0
2689	GH2	0	-	0	0
2690	GHR	1	DB00052	0	0
2697	GJA1	1	DB01136	1	0
2735	GLI1	0	-	1	1
2736	GLI2	0	-	0	1
2737	GLI3	0	-	0	1
2782	GNB1	1	-	0	0
2784	GNB3	0	-	0	0
2785	GNG3	0	-	0	0
2796	GNRH1	0	-	0	0
2797	GNRH2	0	-	0	0
2810	SFN	0	-	1	0
2811	GP1BA	1	DB05202	0	0
2812	GP1BB	0	-	0	0
2814	GP5	0	-	0	0
2815	GP9	1	DB00468	0	0
2872	MKNK2	1	DB12010	0	0
2892	GRIA3	1	DB00142	0	0
2925	GRPR	0	-	0	0

2934	GSN	1	DB01593	1	0
2997	GYS1	0	-	0	0
2998	GYS2	0	-	0	0
3001	GZMA	0	-	0	0
3002	GZMB	1	DB01017	0	0
3053	SERPIND1	1	DB00407	0	0
3082	HGF	1	DB01109	0	0
3084	NRG1	0	-	0	0
3105	HLA-A	1	DB02740	0	0
3106	HLA-B	1	DB04464	0	0
3107	HLA-C	0	-	0	0
3133	HLA-E	0	-	0	0
3134	HLA-F	0	-	0	0
3135	HLA-G	0	-	0	0
3269	HRH1	1	DB00215	0	0
3274	HRH2	1	DB00272	0	0
3303	HSPA1A	1	-	0	1
3304	HSPA1B	0	-	0	0
3305	HSPA1L	0	-	0	0
3306	HSPA2	1	DB04216	0	0
3310	HSPA6	0	-	0	0
3312	HSPA8	1	DB01254	0	0
3339	HSPG2	1	DB00039	0	0
3356	HTR2A	1	DB00246	0	0
3357	HTR2B	1	DB00216	0	0
3358	HTR2C	1	DB00193	0	0
3360	HTR4	1	DB00604	0	0
3361	HTR5A	1	DB00334	0	0
3362	HTR6	1	DB00246	0	0
3363	HTR7	1	DB00216	0	0
3371	TNC	0	-	0	0
3381	IBSP	0	-	0	0
3439	IFNA1	0	-	0	0
3440	IFNA2	1	DB12773	0	0
3441	IFNA4	0	-	0	0
3442	IFNA5	0	-	0	0
3443	IFNA6	0	-	0	0
3444	IFNA7	0	-	0	0
3445	IFNA8	0	-	0	0
3446	IFNA10	0	-	0	0
3447	IFNA13	0	-	0	0
3448	IFNA14	0	-	0	0
3449	IFNA16	0	-	0	0
3451	IFNA17	0	-	0	0

3452	IFNA21	0	-	0	0
3454	IFNAR1	1	DB00008	0	0
3455	IFNAR2	1	DB00008	0	0
3456	IFNB1	1	DB02379	0	0
3458	IFNG	1	DB01250	0	1
3459	IFNGR1	1	DB00033	0	0
3460	IFNGR2	1	DB00033	0	0
3467	IFNW1	0	-	0	0
3486	IGFBP3	1	DB00523	1	0
3516	RBPJ	0	-	0	0
3552	IL1A	1	DB06372	0	0
3553	IL1B	1	DB01017	0	0
3558	IL2	1	DB00852	0	0
3559	IL2RA	1	DB00004	0	0
3560	IL2RB	1	DB00004	0	0
3561	IL2RG	1	DB00004	0	0
3562	IL3	1	DB01025	0	0
3563	IL3RA	1	DB00020	0	0
3565	IL4	1	DB06560	0	0
3566	IL4R	1	DB05078	0	0
3567	IL5	1	DB01411	0	0
3568	IL5RA	1	DB12023	0	0
3569	IL6	1	DB01404	0	0
3570	IL6R	1	DB06273	0	0
3572	IL6ST	0	-	0	0
3574	IL7	0	-	0	0
3575	IL7R	0	-	0	1
3578	IL9	0	-	0	0
3581	IL9R	0	-	0	0
3586	IL10	1	DB05744	0	0
3587	IL10RA	0	-	0	0
3588	IL10RB	0	-	0	0
3589	IL11	0	-	0	0
3590	IL11RA	1	DB00038	0	0
3592	IL12A	0	-	0	0
3593	IL12B	1	DB02763	0	0
3594	IL12RB1	0	-	0	0
3595	IL12RB2	0	-	0	0
3596	IL13	1	DB05305	0	0
3597	IL13RA1	0	-	0	0
3598	IL13RA2	1	DB05078	0	0
3600	IL15	1	DB01327	0	0
3601	IL15RA	0	-	0	0
3630	INS	1	DB01593	0	0

3656	IRAK2	0	-	0	0
3661	IRF3	0	-	1	0
3663	IRF5	0	-	1	0
3665	IRF7	0	-	0	0
3681	ITGAD	0	-	0	0
3682	ITGAE	0	-	0	0
3687	ITGAX	0	-	0	0
3702	ITK	1	DB02010	0	1
3706	ITPKA	1	DB01863	0	0
3707	ITPKB	0	-	0	0
3714	JAG2	0	-	0	0
3716	JAK1	1	DB02375	0	1
3717	JAK2	1	DB04716	0	1
3718	JAK3	1	DB04716	0	0
3725	JUN	1	DB00570	0	1
3745	KCNB1	1	DB06637	0	0
3784	KCNQ1	1	DB01244	0	0
3806	KIR2DS1	0	-	0	0
3808	KIR2DS3	0	-	0	0
3809	KIR2DS4	0	-	0	0
3810	KIR2DS5	0	-	0	0
3822	KLRC2	0	-	0	0
3823	KLRC3	0	-	0	0
3908	LAMA2	0	-	0	0
3909	LAMA3	1	DB06245	0	0
3910	LAMA4	0	-	0	0
3911	LAMA5	1	DB06245	0	0
3912	LAMB1	1	DB06245	0	0
3913	LAMB2	0	-	0	0
3914	LAMB3	0	-	0	0
3915	LAMC1	1	DB06245	0	0
3918	LAMC2	0	-	0	0
3952	LEP	0	-	0	0
3953	LEPR	1	DB05098	0	0
3955	LFNG	0	-	0	0
3972	LHB	0	-	0	0
3976	LIF	0	-	0	0
3977	LIFR	0	-	1	0
3983	ABLIM1	0	-	0	0
3991	LIPE	0	-	0	0
4049	LTA	1	DB00005	0	0
4050	LTB	0	-	0	0
4055	LTBR	0	-	0	0
4091	SMAD6	0	-	0	0

4092	SMAD7	0	-	0	0
4149	MAX	0	-	1	0
4194	MDM4	0	-	0	1
4215	MAP3K3	1	DB12010	0	0
4217	MAP3K5	0	-	0	0
4242	MFNG	0	-	0	1
4254	KITLG	0	-	0	0
4261	CIITA	0	-	0	0
4286	MITF	0	-	0	1
4313	MMP2	1	DB00786	0	0
4342	MOS	0	-	0	1
4352	MPL	1	DB05332	0	1
4609	MYC	1	DB08813	0	1
4616	GADD45B	0	-	1	0
4688	NCF2	1	DB00514	0	0
4689	NCF4	1	DB00514	0	0
4747	NEFL	0	-	0	0
4763	NF1	0	-	1	0
4772	NFATC1	1	DB00852	0	0
4773	NFATC2	0	-	1	0
4775	NFATC3	0	-	0	0
4776	NFATC4	0	-	0	0
4790	NFKB1	1	DB01041	1	0
4792	NFKBIA	1	DB00945	0	0
4800	NFYA	0	-	0	0
4801	NFYB	0	-	0	0
4802	NFYC	0	-	0	0
4846	NOS3	1	DB00125	0	0
4862	NPAS2	0	-	1	0
4923	NTSR1	0	-	0	0
4982	TNFRSF11B	0	-	0	0
5008	OSM	0	-	0	0
5021	OXTR	1	DB00107	0	0
5104	SERPINA5	1	DB00013	0	0
5132	PDC	1	DB04272	0	0
5136	PDE1A	1	DB00622	0	0
5137	PDE1C	1	DB00201	0	0
5139	PDE3A	1	DB00235	0	0
5140	PDE3B	1	DB01640	0	0
5153	PDE1B	1	DB00622	0	0
5154	PDGFA	0	-	0	1
5155	PDGFB	1	DB06325	0	1
5187	PER1	1	DB09060	0	0
5216	PFN1	1	DB07908	1	0

5217	PFN2	1	DB02078	0	0
5228	PGF	1	DB08885	0	0
5265	SERPINA1	1	DB01593	0	0
5305	PIP4K2A	0	-	0	0
5319	PLA2G1B	1	DB00795	0	0
5320	PLA2G2A	1	DB00328	1	0
5321	PLA2G4A	1	DB00588	0	0
5322	PLA2G5	0	-	0	0
5329	PLAUR	1	DB00009	0	0
5330	PLCB2	0	-	0	0
5331	PLCB3	0	-	1	0
5332	PLCB4	0	-	0	0
5337	PLD1	1	DB00122	1	0
5345	SERPINF2	1	DB08888	0	0
5347	PLK1	1	DB06897	1	1
5371	PML	1	DB01169	1	1
5443	POMC	1	DB00836	0	0
5494	PPM1A	0	-	1	0
5495	PPM1B	0	-	0	0
5506	PPP1R3A	0	-	0	0
5507	PPP1R3C	0	-	0	0
5509	PPP1R3D	0	-	0	0
5520	PPP2R2A	1	DB02506	0	0
5521	PPP2R2B	0	-	0	0
5522	PPP2R2C	0	-	1	0
5536	PPP5C	1	DB00171	0	0
5562	PRKAA1	1	DB00131	1	0
5563	PRKAA2	1	DB00945	1	0
5564	PRKAB1	1	DB00131	0	0
5565	PRKAB2	1	DB00131	0	0
5571	PRKAG1	1	DB00945	0	0
5573	PRKAR1A	1	DB01790	1	0
5575	PRKAR1B	0	-	0	0
5576	PRKAR2A	1	DB05798	0	0
5577	PRKAR2B	1	DB02527	0	0
5580	PRKCD	1	DB04376	1	0
5581	PRKCE	1	DB06064	1	1
5583	PRKCH	0	-	0	0
5588	PRKCQ	1	DB02010	0	0
5591	PRKDC	1	DB00201	0	0
5592	PRKG1	0	-	0	0
5593	PRKG2	1	DB12010	0	0
5598	MAPK7	1	DB12010	0	0
5600	MAPK11	1	DB05157	0	0

5603	MAPK13	1	DB05157	0	0
5606	MAP2K3	1	DB12010	0	0
5607	MAP2K5	1	DB12010	0	0
5608	MAP2K6	1	DB12010	0	0
5617	PRL	0	-	0	0
5618	PRLR	1	DB00052	0	0
5644	PRSS1	1	DB01665	0	0
5645	PRSS2	1	DB01805	0	0
5646	PRSS3	1	DB02308	0	0
5649	RELN	0	-	0	0
5663	PSEN1	0	-	0	0
5664	PSEN2	0	-	0	0
5720	PSME1	1	DB09130	0	0
5721	PSME2	0	-	0	0
5724	PTAFR	1	DB02261	0	0
5726	TAS2R38	0	-	0	0
5731	PTGER1	1	DB00297	0	0
5733	PTGER3	1	DB00905	0	0
5737	PTGFR	1	DB00287	0	0
5770	PTPN1	1	DB01133	1	0
5778	PTPN7	0	-	0	0
5787	PTPRB	1	DB06989	0	0
5792	PTPRF	0	-	0	0
5795	PTPRJ	0	-	1	0
5797	PTPRM	0	-	0	0
5801	PTPRR	0	-	0	0
5817	PVR	1	DB03203	0	0
5818	NECTIN1	0	-	0	0
5819	NECTIN2	0	-	0	0
5829	PXN	0	-	0	0
5834	PYGB	1	DB00114	0	0
5836	PYGL	1	DB00114	0	0
5837	PYGM	1	DB00114	0	0
5871	MAP4K2	1	DB12010	0	0
5914	RARA	1	DB00210	0	1
5923	RASGRF1	0	-	0	0
5924	RASGRF2	0	-	0	0
5925	RB1	1	DB00030	1	0
5933	RBL1	0	-	1	0
5934	RBL2	0	-	1	0
5970	RELA	1	DB02836	0	0
5986	RFNG	0	-	0	0
5993	RFX5	0	-	0	0
5994	RFXAP	0	-	0	0

5998	RGS3	0	-	0	0
6195	RPS6KA1	1	DB04751	0	0
6196	RPS6KA2	0	-	1	0
6197	RPS6KA3	1	DB00945	0	0
6198	RPS6KB1	0	-	0	0
6199	RPS6KB2	0	-	0	0
6300	MAPK12	1	DB02482	0	0
6382	SDC1	0	-	0	0
6383	SDC2	1	DB00020	0	0
6385	SDC4	0	-	0	0
6387	CXCL12	1	DB06822	1	0
6416	MAP2K4	0	-	1	0
6422	SFRP1	0	-	1	0
6423	SFRP2	0	-	1	0
6424	SFRP4	1	DB00606	1	0
6425	SFRP5	0	-	1	0
6494	SIPA1	0	-	0	0
6500	SKP1	1	DB01750	0	0
6558	SLC12A2	1	DB00761	0	0
6585	SLIT1	0	-	0	0
6647	SOD1	1	DB00668	0	0
6667	SP1	0	-	0	0
6688	SPI1	0	-	1	1
6696	SPP1	0	-	0	0
6772	STAT1	0	-	1	0
6773	STAT2	0	-	0	0
6774	STAT3	1	DB05959	1	1
6775	STAT4	0	-	0	0
6776	STAT5A	0	-	1	0
6777	STAT5B	1	DB01254	0	0
6778	STAT6	0	-	0	0
6788	STK3	1	DB12010	0	0
6789	STK4	0	-	0	0
6865	TACR2	1	DB06660	0	0
6868	ADAM17	1	DB06943	0	0
6869	TACR1	1	DB00673	0	0
6870	TACR3	1	DB04872	0	0
6885	MAP3K7	0	-	0	1
6915	TBXA2R	1	DB01207	0	0
6932	TCF7	0	-	0	0
6934	TCF7L2	0	-	1	0
7027	TFDP1	0	-	0	0
7035	TFPI	1	DB00036	0	0
7039	TGFA	0	-	0	0

7044	LEFTY2	0	-	1	0
7057	THBS1	0	-	1	0
7058	THBS2	0	-	0	0
7059	THBS3	0	-	0	0
7060	THBS4	0	-	0	0
7070	THY1	0	-	1	0
7074	TIAM1	0	-	0	0
7097	TLR2	1	DB00045	0	0
7098	TLR3	0	-	0	0
7100	TLR5	0	-	0	0
7124	TNF	1	DB00005	0	0
7143	TNR	0	-	0	0
7148	TNXB	0	-	0	0
7173	TPO	1	DB00389	0	0
7201	TRHR	1	DB09421	0	0
7248	TSC1	0	-	1	0
7252	TSHB	0	-	0	0
7253	TSHR	1	DB00024	0	0
7297	TYK2	1	DB04716	0	0
7299	TYR	1	DB00157	0	0
7412	VCAM1	1	DB00898	0	0
7422	VEGFA	1	DB00112	1	0
7423	VEGFB	1	DB08885	0	0
7424	VEGFC	0	-	0	0
7448	VTN	1	DB00054	0	0
7450	VWF	1	DB00025	0	0
7465	WEE1	1	DB04608	0	0
7525	YES1	1	DB01254	0	1
8027	STAM	0	-	0	0
8074	FGF23	1	DB14012	0	0
8302	KLRC4	0	-	0	0
8394	PIP5K1A	0	-	0	0
8395	PIP5K1B	0	-	0	0
8396	PIP4K2B	1	DB12010	0	0
8398	PLA2G6	1	DB01103	0	0
8399	PLA2G10	1	DB05737	0	0
8408	ULK1	1	DB12010	0	0
8454	CUL1	0	-	1	0
8482	SEMA7A	0	-	0	0
8569	MKNK1	1	DB12010	0	0
8600	TNFSF11	1	DB00480	0	0
8625	RFXANK	0	-	0	0
8633	UNC5C	0	-	1	0
8646	CHRD	0	-	0	0

8650	NUMB	0	-	1	0
8651	SOCS1	0	-	1	0
8681	JMJD7-PLA2G4B	0	-	0	0
8740	TNFSF14	0	-	0	0
8743	TNFSF10	0	-	0	0
8764	TNFRSF14	0	-	0	0
8771	TNFRSF6B	0	-	0	0
8792	TNFRSF11A	1	DB05959	0	0
8817	FGF18	0	-	0	0
8822	FGF17	0	-	0	0
8823	FGF16	0	-	0	0
8835	SOCS2	0	-	0	0
8837	CFLAR	0	-	0	1
8850	KAT2B	1	DB01992	0	0
8863	PER3	0	-	0	0
8864	PER2	0	-	1	0
8874	ARHGEF7	0	-	0	0
8877	SPHK1	0	-	0	0
8915	BCL10	0	-	1	0
8945	BTRC	0	-	0	0
9002	F2RL3	1	DB11300	0	0
9021	SOCS3	0	-	1	0
9047	SH2D2A	0	-	0	0
9133	CCNB2	0	-	0	1
9180	OSMR	0	-	0	0
9181	ARHGEF2	0	-	0	1
9241	NOG	0	-	0	0
9252	RPS6KA5	0	-	0	0
9253	NUMBL	0	-	0	0
9350	CER1	0	-	0	0
9353	SLIT2	0	-	1	0
9423	NTN1	0	-	0	0
9451	EIF2AK3	0	-	0	0
9470	EIF4E2	0	-	0	0
9541	CIR1	0	-	0	0
9542	NRG2	0	-	0	0
9575	CLOCK	0	-	0	0
9612	NCOR2	0	-	0	0
9655	SOCS5	0	-	0	0
9672	SDC3	0	-	0	0
9693	RAPGEF2	0	-	0	0
9706	ULK2	1	DB12010	0	0
9794	MAML1	0	-	0	0
9863	MAGI2	0	-	0	0

9899	SV2B	0	-	0	0
9900	SV2A	1	DB01202	0	0
9965	FGF19	1	DB01109	0	0
9978	RBX1	0	-	0	0
10093	ARPC4	1	DB08235	0	0
10094	ARPC3	1	DB08235	0	0
10095	ARPC1B	1	DB08235	0	0
10109	ARPC2	1	DB08235	0	0
10125	RASGRP1	0	-	0	0
10154	PLXNC1	0	-	1	0
10197	PSME3	0	-	0	0
10235	RASGRP2	0	-	0	0
10254	STAM2	0	-	0	0
10297	APC2	0	-	0	0
10319	LAMC3	0	-	0	0
10411	RAPGEF3	0	-	0	0
10452	TOMM40	0	-	0	0
10468	FST	1	DB01666	0	0
10505	SEMA4F	0	-	0	0
10507	SEMA4D	0	-	0	0
10509	SEMA4B	0	-	0	0
10552	ARPC1A	0	-	0	0
10603	SH2B2	0	-	0	0
10637	LEFTY1	0	-	1	0
10666	CD226	0	-	0	0
10683	DLL3	0	-	0	0
10718	NRG3	0	-	0	0
10725	NFAT5	0	-	0	0
10788	IQGAP2	0	-	1	0
10800	CYSLTR1	1	DB00471	0	0
10870	HCST	0	-	0	0
10892	MALT1	0	-	0	1
10912	GADD45G	0	-	1	0
11009	IL24	0	-	1	0
11069	RAPGEF4	0	-	0	0
11072	DUSP14	0	-	0	0
11186	RASSF1	0	-	1	1
11197	WIF1	0	-	1	0
11213	IRAK3	1	DB12010	0	0
11221	DUSP10	0	-	0	0
11317	RBPJL	0	-	0	0
22798	LAMB4	0	-	0	0
22885	ABLIM3	0	-	0	0
22926	ATF6	1	DB00852	0	0

22987	SV2C	0	-	0	0
23220	DTX4	0	-	0	0
23236	PLCB1	0	-	0	0
23291	FBXW11	0	-	0	0
23308	ICOSLG	0	-	0	0
23385	NCSTN	0	-	0	0
23433	RHOQ	0	-	0	0
23529	CLCF1	0	-	0	0
25780	RASGRP3	0	-	0	0
25945	NECTIN3	0	-	0	0
25989	ULK3	1	DB12010	0	0
26230	TIAM2	0	-	0	0
26279	PLA2G2D	1	DB03017	0	0
26281	FGF20	0	-	0	0
26291	FGF21	0	-	0	0
27006	FGF22	0	-	0	0
27035	NOX1	1	DB09140	0	0
27330	RPS6KA6	1	DB12010	1	0
28514	DLL1	0	-	0	0
29949	IL19	0	-	0	0
29984	RHOD	0	-	0	0
30814	PLA2G2E	1	DB00233	0	0
30837	SOCS7	0	-	0	0
50487	PLA2G3	0	-	0	0
50508	NOX3	0	-	0	0
50509	COL5A3	0	-	0	0
50604	IL20	0	-	0	0
50615	IL21R	0	-	0	0
50616	IL22	0	-	0	0
50831	TAS2R3	0	-	0	0
50832	TAS2R4	0	-	0	0
50833	TAS2R16	1	DB01032	0	0
50834	TAS2R1	0	-	0	0
50835	TAS2R9	0	-	0	0
50836	TAS2R8	0	-	0	0
50837	TAS2R7	0	-	0	0
50838	TAS2R13	0	-	0	0
50839	TAS2R10	0	-	0	0
50840	TAS2R14	0	-	0	0
50848	F11R	0	-	0	0
50855	PARD6A	0	-	0	0
51107	APH1A	1	DB05171	0	0
51176	LEF1	1	DB00903	0	1
51206	GP6	0	-	0	0

51422	PRKAG2	1	DB00945	0	0
51561	IL23A	1	DB05459	0	0
51701	NLK	0	-	0	0
51764	GNG13	0	-	0	0
53632	PRKAG3	1	DB00945	0	0
53832	IL20RA	0	-	0	0
53833	IL20RB	0	-	0	0
53944	CSNK1G1	1	DB08325	0	0
54106	TLR9	1	DB00608	0	0
54205	CYCS	1	DB01017	0	0
54429	TAS2R5	0	-	0	0
54472	TOLLIP	0	-	0	0
54567	DLL4	0	-	0	0
54910	SEMA4C	0	-	0	0
55534	MAML3	0	-	0	0
55740	ENAH	0	-	0	0
55844	PPP2R2D	0	-	0	0
55851	PSENEN	1	DB05171	0	0
56034	PDGFC	0	-	0	0
56413	LTB4R2	1	DB06248	0	0
56832	IFNK	0	-	0	0
56848	SPHK2	0	-	0	0
56998	CTNNBIP1	0	-	1	0
57105	CYSLTR2	1	DB00716	0	0
57154	SMURF1	0	-	0	1
57369	GJD2	0	-	0	0
57680	CHD8	0	-	0	0
57715	SEMA4G	0	-	0	0
58494	JAM2	0	-	0	0
58985	IL22RA1	0	-	0	0
59067	IL21	0	-	0	0
59343	SENP2	0	-	0	0
63923	TNN	0	-	0	0
64109	CRLF2	0	-	0	1
64218	SEMA4A	0	-	0	0
64221	ROBO3	0	-	0	0
64321	SOX17	0	-	0	0
64600	PLA2G2F	0	-	0	0
64750	SMURF2	0	-	0	0
64840	PORCN	0	-	0	0
79139	DERL1	0	-	0	0
79660	PPP1R3B	0	-	0	0
79837	PIP4K2C	1	DB12010	0	0
80301	PLEKHO2	0	-	0	0

80310	PDGFD	1	DB05139	0	0
80319	CXXC4	0	-	1	0
80824	DUSP16	0	-	0	0
80834	TAS1R2	1	DB00168	0	0
80835	TAS1R1	0	-	0	0
81579	PLA2G12A	0	-	0	0
81607	NECTIN4	0	-	0	1
83439	TCF7L1	0	-	0	0
83700	JAM3	0	-	0	0
83756	TAS1R3	1	DB00168	0	0
84134	TOMM40L	0	-	0	0
84433	CARD11	0	-	0	1
84441	MAML2	0	-	0	1
84448	ABLIM2	0	-	0	0
84552	PARD6G	0	-	0	0
84612	PARD6B	0	-	0	0
84647	PLA2G12B	0	-	0	0
84867	PTPN5	0	-	0	0
85407	NKD1	0	-	0	0
85409	NKD2	0	-	0	0
85417	CCNB3	0	-	0	0
85477	SCIN	0	-	0	0
85480	TSLP	0	-	1	0
90249	UNC5A	0	-	1	0
113878	DTX2	0	-	0	0
115653	KIR3DL3	0	-	0	0
115727	RASGRP4	0	-	0	0
116379	IL22RA2	0	-	0	0
117157	SH2D1B	0	-	0	0
122011	CSNK1A1L	0	-	0	0
122809	SOCS4	0	-	0	0
128239	IQGAP3	0	-	0	0
131873	COL6A6	0	-	0	0
137970	UNC5D	0	-	1	0
145957	NRG4	0	-	0	0
149233	IL23R	0	-	0	0
150084	IGSF5	0	-	0	0
151636	DTX3L	0	-	0	0
163702	IFNLR1	0	-	0	0
196403	DTX3	0	-	0	0
196883	ADCY4	0	-	0	0
200576	PIKFYVE	0	-	0	0
219699	UNC5B	0	-	1	0
253314	EIF4E1B	0	-	0	0

259285	TAS2R39	0	-	0	0
259286	TAS2R40	0	-	0	0
259287	TAS2R41	0	-	0	0
259289	TAS2R43	0	-	0	0
259290	TAS2R31	0	-	0	0
259291	TAS2R45	0	-	0	0
259292	TAS2R46	0	-	0	0
259294	TAS2R19	0	-	0	0
259295	TAS2R20	0	-	0	0
259296	TAS2R50	0	-	0	0
260425	MAGI3	0	-	0	0
282616	IFNL2	0	-	0	0
282617	IFNL3	0	-	0	0
282618	IFNL1	0	-	0	0
284217	LAMA1	1	DB06245	0	0
338376	IFNE	0	-	0	0
338398	TAS2R60	0	-	0	0
345456	PFN3	0	-	0	0
353164	TAS2R42	0	-	0	0
375189	PFN4	0	-	0	0
375790	AGRN	0	-	0	1
646048	-	0	-	0	0
646821	-	0	-	0	0
648921	-	0	-	0	0
649853	-	0	-	0	0
650832	-	0	-	0	0
652346	-	0	-	0	0
652671	-	0	-	0	0
653361	NCF1	1	DB00514	0	0
653888	-	0	-	0	0
728622	SKP1P2	0	-	0	0
731751	-	0	-	0	0
100133672	-	0	-	0	0
100137049	PLA2G4B	0	-	0	0

Table S3. Gene information of TGL.

Gene name	Drug-target (1: True, 0: False)	Drug ID (-: unknown)	Tumor suppressor (1: True, 0: False)	Oncogene (1: True, 0: False)
TAX	0	-	0	1
Stimuli2	0	-	0	0
IFN	0	-	0	0
IL2RAT	0	-	0	0
Cytoskeleton_signaling	0	-	0	0
PDGF	0	-	0	0
Stimuli	0	-	0	0
sFas	0	-	0	0
A20	0	-	1	0
FasT	0	-	0	0
NFAT	0	-	0	0
TBET	0	-	0	0
Proliferation	0	-	0	0
P27	0	-	0	0
P2	1	DB04137	0	0
SPHK1	0	-	0	0
PLCG1	0	-	0	0
MEK	0	-	0	0
STAT3	1	DB05959	1	1
PDGFR	1	-	0	1
S1P	0	-	0	0
JAK	0	-	0	1
RANTES	0	-	0	0
GZMB	1	DB01017	0	0
CREB	1	-	0	1
IL2	1	DB00852	0	0
TRADD	0	-	0	0
IL2RA	1	DB00004	0	0
BclxL	0	-	0	0
SMAD	0	-	0	0
GPCR	0	-	0	0
CTLA4	1	DB06186	0	0
GRB2	1	DB00061	0	0
TCR	0	-	0	0
ZAP70	1	DB02010	0	0
IFNG	1	DB01250	0	1
TNF	1	DB00005	0	0
FasL	0	-	0	0
FYN	1	DB01254	0	1
SOCS	0	-	0	0
ERK	0	-	1	0

PI3K	1	-	0	1
RAS	0	-	0	0
IAP	0	-	0	0
NFKB	1	-	0	0
TPL2	0	-	1	1
IFNGT	0	-	0	0
IL2RBT	0	-	0	0
Apoptosis	0	-	0	0
Ceramide	0	-	0	0
Fas	0	-	1	1
FLIP	0	-	0	1
DISC	0	-	0	0
MCL1	1	DB09401	0	1
IL2RB	1	DB00004	0	0
LCK	1	DB01254	0	1
CD45	0	-	1	0
GAP	1	DB02263	0	0
IL15	1	DB01327	0	0
BID	0	-	0	0
Caspase	0	-	0	0
